# Healthcare Access for Patients With Inflammatory Bowel Disease in the United States: A Survey by the Crohn’s & Colitis Foundation

**DOI:** 10.1093/ibd/izae237

**Published:** 2024-10-08

**Authors:** Ariel A Jordan, Shubha Bhat, Tauseef Ali, Sarah R Brunskill, Nancy A Clusen, Ross M Maltz, Ced Moise, Xiaofan Sun, Harry J Thomas, Cassie Ray, Mary Harkins-Schwarz, Orna G Ehrlich

**Affiliations:** Department of Internal Medicine, University of Michigan, Ann Arbor, MI, USA; Department of Pharmacy, Cleveland Clinic Foundation, Cleveland, OH, USA; Department of Digestive Disease Institute, Cleveland Clinic Foundation, Cleveland, OH, USA; Crohn’s and Colitis Center, SSM Health St. Anthony Hospital Digestive Care, Oklahoma City, OK, USA; Mathematica, Princeton, NJ, USA; Mathematica, Princeton, NJ, USA; Division of Pediatric Gastroenterology, Hepatology and Nutrition, Nationwide Children’s Hospital, Columbus, OH, USA; Department of Pediatrics, The Ohio State Wexner Medical Center, Columbus, OH, USA; Mathematica, Princeton, NJ, USA; Mathematica, Princeton, NJ, USA; Austin Gastroenterology, Austin, TX, USA; Crohn’s & Colitis Foundation, New York, NY, USA; Crohn’s & Colitis Foundation, New York, NY, USA; Crohn’s & Colitis Foundation, New York, NY, USA

**Keywords:** inflammatory bowel disease, insurance, barriers to care, Crohn’s disease, ulcerative colitis

## Abstract

**Background:**

A prior survey disseminated in 2017 identified that healthcare access barriers exist and significantly affect patients with inflammatory bowel disease (IBD). We sought to identify, through an updated survey, the healthcare access barriers that patients continue to face, with a focus on socioeconomic factors and patient awareness of resources to navigate existing barriers.

**Methods:**

A 52-question online survey evaluating (1) access to healthcare professionals, medications, and procedures; (2) associated financial challenges; and (3) patient awareness of education and advocacy tools to navigate IBD care barriers, was disseminated through multiple channels to IBD patients and their caregivers.

**Results:**

Of the 2281 completed responses, patients on advanced specialty medications, younger than 65 years of age, or on employer insurance experienced significantly greater issues with insurance barriers to accessing medications and coverage of medically necessary tests/treatments. Patients who live in areas of concentrated poverty were more likely to experience poor health outcomes when subjected to step therapy compared to patients who did not. Additionally, patients were more likely to experience one or more financial barriers or trade-offs if the patient used an advanced specialty medicine or lived in an area with concentrated poverty.

**Conclusions:**

While there have been significant and numerous advancements in IBD treatments, patients with IBD continue to experience barriers to healthcare access and treatment and financial struggles. Ongoing awareness and advocacy efforts focused on healthcare system reform and related policies to further minimize care disparities and barriers remain vital.

## Graphical Abstract

**Graphical Abstract:**
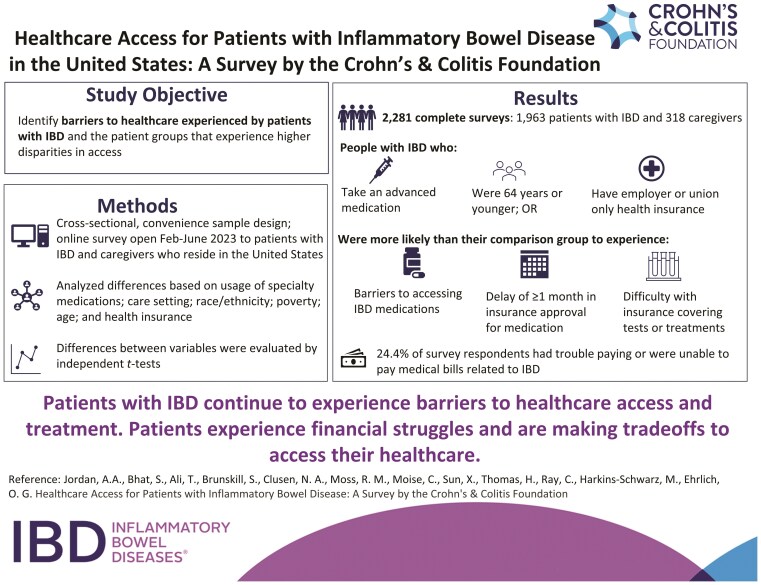

**Key Messages**

**What is already known?**

Significant access to care barriers exists for people with inflammatory bowel disease (IBD), resulting in worse health outcomes and lower quality of life.

**What is new here?**

This study provided updated information on specific socioeconomic barriers to treatments and testing and addressed the impact of the social determinants of health and recent legislative reform on care access.

**How can this study help patient care?**

Findings highlight the current care challenges people with IBD face and emphasize the importance of advocacy among healthcare professionals and patient support organizations to reduce access barriers.

## Introduction

Inflammatory bowel disease (IBD) is a chronic, immune-mediated inflammatory disease requiring long-term medical management, often with costly medications that require regular follow-up visits and frequent disease activity monitoring, which leads to expensive and often prohibitive cost of care burden for patients.^1^ In 2017, the Crohn’s & Colitis Foundation (Foundation) healthcare access survey of 3608 patients with IBD found that many patients struggled with financial barriers related to care due to insurance limitations and utilized emergency services frequently due to lack of timely access to care.^2^ Since that survey was published, there have been advances in knowledge of the epidemiology of IBD,^3,4^ approach to IBD management,^2,5^ increased medication options, and policy changes, all of which have impacted patients’ IBD management and access to care.^6^

The global incidence and prevalence of IBD have increased substantially over the last several decades, highlighting IBD as a disease that affects diverse patient populations.^3,7,8^ Simultaneously, there is more awareness of the significant socioeconomic disparities that impact patients with IBD, resulting in varied healthcare outcomes, a concept known as the social determinants of health (SDOH).^4,7^ A 2020 study by Nguyen et al.^9^ evaluated barriers due to SDOH in patients with IBD and found that 1 in 8 patients struggled with at least one socioeconomic hardship including food insecurity, inadequate social support, or cost-related medication nonadherence. Healthcare costs for IBD have also significantly increased in the last decade, with cost of care and out-of-pocket costs being 2-3 times greater for patients with IBD compared to non-IBD patients, with most spending being driven by biologics and emergency services utilization.^1^ As a result, despite multiple recently FDA-approved medications for adult patients with IBD, including biosimilars,^10^ payers in the United States continue to mandate fail first protocols or step therapy. Fail-first protocols consist of insurance mandating that patients fail a therapy first before being able to initiate the healthcare professional’s preferred medication. Policies like this require patients to start low-cost medications which are often inadequate treatments for their IBD.^11^ As of April 2024, 37 states have passed legislation to protect patients from step therapy or “fail-first” protocols to ensure a clearer, transparent appeals process for those with state-regulated insurance.^12^ The Foundation and other patient-focused organizations have continued to advocate for this legislation to be passed by every state in the country and for a similar federal bill to protect patients who have insurance that is regulated by the federal government.

Another significant advance since the initial access to care survey is the shift in IBD management to multidisciplinary models,^5^ which has shown reduced unplanned care, improved patient disease activity, and overall improved quality of life.^5,13,14^ Similar multidisciplinary models are rapidly being adopted by many adult and pediatric academic medical centers which is beneficial for patients, but not available in all treatment settings.^15^

Inadequate access to healthcare significantly impacts patients’ quality of life, thus there is a need to characterize the current landscape and barriers, which can guide advocacy and reform focus and efforts. We conducted an updated patient survey in 2023 to assess patients’ access to IBD prescription medications, treatments, and testing; and cost-sharing and financial trade-offs. The survey also assessed patients’ awareness of protections relating to insurance and awareness of resources to help manage and advocate for their care. Finally, the survey assessed confidence in the ability to navigate health insurance challenges. We hypothesized there would be differences in access to care by different population groups (eg, patients who take advanced specialty medications, receive care in an academic setting, and demographic characteristics, including age, race, ethnicity, poverty, and health insurance).

## Materials and Methods

### Design

The survey (Supplementary Data) consisted of 52 questions structured across 7 parts: access to healthcare professionals; use of and delays in medications, tests, and treatments; financial barriers impacting IBD healthcare; use of exclusive enteral nutrition; awareness of step therapy reform legislation; and awareness and use of patient education and advocacy tools and resources. Disease characteristics and survey participant demographics were collected.

The Foundation developed the survey in collaboration with Mathematica and a stakeholder group comprised of medical advisors, patients with IBD, and caregivers. We designed one survey with 2 distinct parts: (1) a patient version and (2) an adapted proxy caregiver version. To determine which version of the survey a respondent should receive, we incorporated a decision tree question at the beginning of the survey to identify the respondent as either a patient or a caregiver. This decision tree then applied programmed skip logic to automatically direct them to the appropriately worded set of questions. At the end of the patient version (question I12), respondents were asked if they were the primary caregiver for someone with IBD and, if so, if they would like to take the survey again on their behalf. At the end of the caregiver version (question C_I12), respondents were asked if they would like to take the survey again on behalf of another person with IBD for which they care.

Questions were modified from existing survey instruments, including the National Health Interview Survey, Consumer Assessment of Healthcare Provider Surveys, and the Kaiser Family Foundation/Los Angeles Times Survey of Adults with Employer-Sponsored Health Insurance.^16-19^ Additionally, the research team developed questions when a gap was identified. The draft survey instrument was pilot-tested to assess (1) survey flow, ease, and length; (2) comprehension, particularly for new or heavily adapted questions; and (3) the ability of the patient and caregiver to complete their versions of the survey. Twelve volunteer participants (6 patients with IBD and 6 caregivers) participated in the pilot across 2 rounds of data collection. The survey was revised based on pilot testing and expert opinion from Mathematica.

### Participants

The survey was open to adults with IBD and caregivers who resided in the United States and US territories (American Samoa, Guam, the Northern Mariana Islands, Puerto Rico, and the US Virgin Islands). IBD diagnosis was self-reported. Caregivers for minors and adults with IBD were allowed to participate and respond on behalf of the patient. The following criteria were used to determine survey eligibility and inclusion in the final dataset: (1) the patient resided in the United States or one of the 5 US territories; (2) caregivers who responded were intimately familiar with patients’ current IBD healthcare needs, care, and medications (those who answered “not at all familiar” or only “slightly familiar” were excluded); (3) the respondent met the age at which a person gains the legal status of adulthood for his or her state of residence (also known as age of majority, typically age 18 or older); and (4) respondent completed the majority of questions up to and including question #I9 (education; this enabled us to limit the missing data within key demographic variables).

### Procedure

The survey was conducted via an online survey platform (SurveyMonkey; San Mateo, CA, United States) and fielded from February through June 2023 (13 weeks). The survey was only available in English and open to anyone who accessed the survey link (convenience sampling approach). The Foundation disseminated recruitment communications through social media, constituent listservs (including patients, caregivers, and healthcare professionals), Foundation medical advisory committee members, regional patient and caregiver education programs, and partner networks.

To reach patients unaffiliated with the Foundation and other patient groups, the Foundation utilized targeted paid social media ads, conducted additional outreach from Foundation chapter offices located in communities with significant non-White populations, and partnered with external organizations (eg, United Ostomy Association, Improve Care Now Network, South Asian IBD Alliance, and Girls with Guts) with reach to non-Foundation connected and/or non-White patients and caregivers with IBD. All responses were collected anonymously, and no incentive was offered.

### Survey Completion Rate

Of the respondents who began the survey (4351, accounting for dual respondent split cases), 2281 completed it, resulting in a 52.4% completion rate among known respondents.

### Ethical Considerations

The study was approved by the Advarra Institutional Review Board and considered exempt from IRB oversight under 45 CFR 46.104(d)(2). Consent was implied if the participant proceeded with the survey.

### Study Variables

We examined, in the prior 12 months, to what extent respondents experienced difficulties related to care for their IBD using the following variables: medication access (eg, insurance would not cover a medication that was prescribed); medication delays (eg, waited one month or longer for insurance to approve medication); coverage for tests or treatments (eg, insurance never or only sometimes approved a test or treatment, such as a calprotectin stool test); step therapy mandates (eg, insurance required patient try a different medication other than the medication prescribed by their healthcare professional); financial barriers to care (eg, in order to pay their healthcare or insurance costs related to their IBD, patient or someone else in the family cut expenses, increased their debt, or increased their income); financial barriers to obtaining medication (eg, did not get medication to save costs); and use of copay programs (eg, received a discount on a medication, either through a coupon, copay card, drug company patient assistance program, or some other kind of medication discount). Complete variable definitions are shown in Supplementary Table S1.

We analyzed whether there were differences for each of the questions above by the following population groups: advanced specialty medications (patient takes a biologic therapy, biosimilar, or targeted synthetic small molecule medication compared to patients that do not take one of these medications); academic setting (patient receives IBD healthcare in an academic setting compared to patients who do not receive care in an academic setting); White, non-Hispanic compared to patients who do not identify as White, non-Hispanic; poverty (patient resides in area of concentrated poverty compared to patients who do not reside in an area of concentrated poverty); age (64 years or younger compared to 65 years or older); and health insurance (patient has employer or union-based health insurance only compared to patient has Medicare only). Population definitions and analysis comparison groups are shown in Supplementary Table S2.

To create a categorization of poverty (patient lives in an area of concentrated poverty), we used the Census Bureau’s benchmark definition of “high poverty areas” (census tracts with poverty rates of 20% or more).^20,21^ High poverty area estimates were derived from 2018 to 2022 American Community Survey (ACS) 5-year estimates,^22^ which provide poverty data for the population for whom poverty status is determined. We utilized ACS data to determine the percentage of the population living in poverty based on the respondent’s reported 5-digit ZIP code (question I11). “Poverty area” was defined as an area with a poverty rate of 20% or more and “not a poverty area” as an area with a poverty rate of 19.9% or less.

### Statistical Analysis

Statistical analyses were performed using SAS/STAT^®^ (version 9.4) statistical software (SAS Institute, Cary, NC, United States). A *P* value <.05 was considered to be statistically significant.^23^

#### Analysis inclusion criteria

Following best practices, the data were examined to determine eligibility, out-of-bound responses, skip pattern errors, and missing data. Data errors were programmatically cleaned and corrected following predetermined survey logic.

Any analysis that did not meet established statistical standards for reporting (eg, insufficient sample size, confidence interval (CI) greater than 20%, or relative CI greater than 120%) was noted as such.

#### Analysis

This paper presents the findings from our analyses. Descriptive statistics were used to describe demographic characteristics and scale mean values. Variables were expressed as absolute number of respondents and percentage of total or means (M) ± SD, where appropriate. Differences between variables were evaluated by independent *t*-tests, and Bonferroni correction for multiple comparisons was applied for results interpretation, considering a corrected *P* ≤.05 as statistically significant. No weights or other adjustments were applied.

### Reporting

This paper is reported following the STROBE statement recommendations.^24^

## Results

### Population

**Figure 1.**
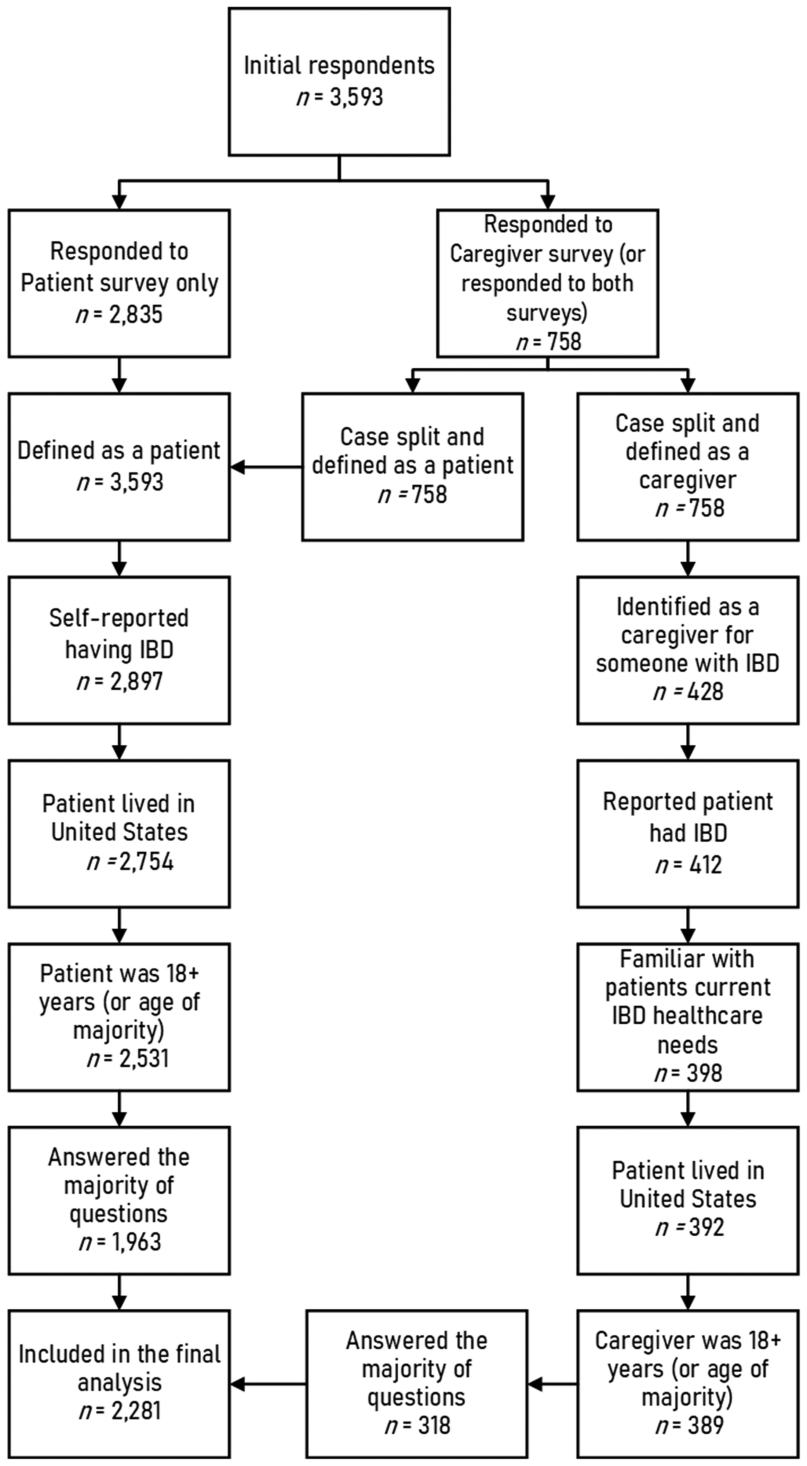
Flow diagram depicting survey eligibility.

The initial data file included 3593 cases; after separating the 2 versions (ie, patient and caregiver) the file included 4351 cases. Specifically, if a respondent was both a patient and a caregiver, we duplicated the case, assigned each case to a respondent type (patient and caregiver), and removed the data from the noncorresponding version. Of these, 2070 cases were considered ineligible (eg, incomplete survey, did not report an IBD diagnosis, lived outside of the United States, under the age of 18 or age of majority, etc.) and 2281 cases were coded as *complete* and used for analysis (Figure 1). Hereafter, the respondent refers to the person with IBD.

Out of the 2281 total cases, 1963 (86.1%) were patients and 318 (13.9%) were caregivers. The majority of the respondents were 26-64 years old (60.2%, *n = *1372), identified as female (68.4%, *n = *1559) and White, non-Hispanic (88.7%, *n = *1957). Most respondents had obtained a college-level degree or higher (68.2%, *n* = 1433), were employed full-time (41.3%, *n = *943), and had health insurance (98.0%, *n = *2204). Among respondents with health insurance, most indicated that they have employer or union-based insurance or Medicare; thus, our analysis focused on 2 groups: employer or union-based insurance only (81.9%, *n *= 1315) and Medicare only (18.1%, *n *= 291; Table 1).

**Table 1.** Demographic characteristics (total *N* = 2281).

Patient characteristics	Percent	*n*
Age		
Birth-25	17.9%	409
26-64	60.2%	1372
65 and older	21.9%	500
Race		
White, non-Hispanic	88.7%	1957
Black, non-Hispanic	3.5%	76
Other, non-Hispanic^a^	4.0%	89
Ethnicity		
Hispanic	3.8%	84
Gender		
Female	68.4%	1559
Male	29.5%	671
Transgender/use a different term	2.1%	48
Education (among patients 18 years and older)		
Less than high school	0.8%	16
High school/some college	27.2%	571
Bachelor’s degree or higher	68.2%	1433
Other	3.9%	81
Employed (respondents could check all that apply)		
Employed, full time	41.3%	943
Employed, part time	10.5%	239
Unemployed	4.2%	96
Homemaker	4.4%	100
Student	13.7%	312
Retired	23.3%	533
Unable to work	7.6%	173
US Region		
Northeast	25.9%	592
Midwest	25.1%	573
South^b^	29.9%	681
West	19.1%	435
Insured	98.0%	2204
Poverty		
Not concentrated poverty (19.9% or less)	93.3%	1975
Concentrated poverty (20.0% or more)	6.8%	143

^a^Other, non-Hispanic respondents identified as American Indian or Alaska Native; Asian; Native Hawaiian or other Pacific Islander; or Other self-identify and did not identify as White, Black, or Hispanic.

^b^To account for participants who reported living in a United States territory (*n* = 3), we included them in the South region.

### Disease and Treatment

Most respondents had Crohn’s disease (66.1%, *n = *1507) and were diagnosed before 2021 (94.2%, *n = *2086) with disease activity relatively split between mild (36.6%, *n = *831), moderate (32.3%, *n = *733), and severe (31.0%, *n = *704). The vast majority of respondents had a place (in person or via telehealth) where they usually received their IBD care (96.5%, *n = *2185). Of respondents who had a regular place for their IBD care, most received their care in a healthcare professional office or clinic (97.5%, *n* = 2128,) or a VA medical center or VA outpatient clinic (1.0%, *n = *20). Among these respondents 36.3% (*n = *779) reported that the healthcare facility was an academic setting (specifically, an academic medical center which provides graduate medical training and research; Table 2).

**Table 2.** Disease and healthcare characteristics IBD Health Access Assessment (Total *N* = 2281).

Characteristics	Percent	*n*
Primary disease type		
Crohn’s disease	66.1%	1507
Ulcerative colitis	30.0%	685
IBD—unclassified	2.4%	55
Unknown or undetermined	1.5%	34
Diagnosis time frame		
Newly diagnosed (diagnosed in 2022 or 2023)	5.8%	129
Not newly diagnosed (diagnosed in 2021 or earlier)	94.2%	2086
Disease activity		
Mild (rarely active, giving symptoms only a few days in past 6 months, or was well in past 6 months, what he/she considers remission or absence of symptoms)	36.6%	831
Moderate (sometimes active, giving symptoms on some days, or occasionally active, giving symptoms 1-2 days a month)	32.3%	733
Severe (constantly active, giving symptoms every day or often active, giving symptoms most days)	31.0%	704
Type of healthcare professional usually go to for IBD care		
Gastroenterologist or NPP/PA in gastroenterologist office	95.8%	2183
Other	4.2%	96
Has a place (in person or via telehealth) where they usually go to receive IBD healthcare		
Yes	96.5%	2185
No	3.5%	79
Type of place goes to for IBD healthcare (among those with a usual place for IBD healthcare)		
Healthcare professional office or clinic	97.5%	2128
VA Medical Center or VA Outpatient clinic	0.9%	20
Hospital emergency room	0.6%	14
Public health clinic, community health center, or tribal clinic	0.3%	7
Some other place	0.6%	13
Goes to an academic setting for IBD healthcare (among respondents whose usual place to receive IBD healthcare is a healthcare professional’s office/clinic or VA medical center/outpatient clinic)		
Yes	36.3	779
No	63.7	1365
Patient uses advanced medication (biologic therapy, biosimilar, or targeted synthetic small molecule to treat their IBD; among respondents prescribed any medication for their IBD in the past 12 months)		
Yes	75.1%	1539
No	24.9%	510

### Insurance Barriers to Care

#### Medication access

In the prior 12 months, 90% (*n = *2051) of respondents were prescribed medication for their IBD, and among these respondents, more than half (56.2%, *n = *1124) experienced problems accessing their medications due to their health insurance (eg, insurance would not cover medication prescribed by their IBD healthcare professional, would not cover the dose or frequency of medication, etc.; Table 3). Of respondents prescribed any medication for IBD in the past 12 months, most were on an advanced specialty medication such as biologic therapy, biosimilar, or targeted synthetic small molecule (75.1%, *n = *1539). Patients were significantly more likely to experience medication access barriers if they took an advanced specialty medication (M* *= 0.61, SD = 24.10, *t*(2300) = −8.38, *P* < .0001), received IBD care at an academic center (M = 0.61, SD = 19.68, *t*(2184) = −3.60, *P* = .002), were 64 years and younger (M = 0.62, SD = 24.88, *t*(2299) = 11.22, *P* < .001), or had employer or union only health insurance (M = 0.60, SD = 19.33, *t*(1426) = −5.00, *P* < .001) when compared to their analysis counterpart (Table 4).

**Table 3.** Experiences of respondents prescribed any medication for their IBD in prior 12 months (Total *N* = 2051).

	Percent	*n*
Have taken corticosteroids for IBD in prior 12 months		
Yes	33.3%	683
No	65.5%	1343
Don’t know	1.2%	25
Have taken advanced medications (biologic therapy, biosimilar, and targeted synthetic small molecule) for IBD in prior 12 months		
Yes	75.1%	1539
No	24.0%	492
Don’t know	1.0%	18
Experienced problems accessing IBD prescription due to health insurance		
Yes	56.2%	1124
No	43.8%	875
Able to get all of their IBD prescription medication in prior 12 months		
Yes	87.1%	1786
No	12.2%	251
Don’t know	0.6%	13
Experienced insurance approval delays of 1 month or longer for IBD medications		
Yes	16.8%	341
No	83.2%	1688
Experienced adverse health outcomes when unable to get or delayed in getting IBD medications		
Yes	69.4%	752
No	30.6%	332
Experienced effects on daily life (work, school, and daily activities) when unable to get or delayed in getting IBD medications		
Yes	48.7%	315
No	51.3%	332

**Table 4.** Independent *t-*test results.

	Advanced specialty medication^a^			Not taking advanced specialty medication			*t*-test	Adjusted *P* value
	*n*	M	SD	*n*	M	SD		
Medication access	1750	0.61	24.1	551	0.41	14.26	−8.38	<.0001
Medication delays	1725	0.19	16.76	563	0.08	6.57	−7.56	<.0001
Coverage for tests or treatments	1590	0.21	16.88	453	0.16	8.29	−2.77	.03
Experienced step therapy and poor health outcomes	352	0.86	9.96	69	0.81	7.08	−0.97	1.00
Financial barriers delay medication	1769	0.16	15.93	591	0.25	11.67	4.15	.000
Use of copay programs	1626	0.70	23.54	549	0.24	11.16	−21.18	<.0001

	Academic setting^b^			Not academic setting			*t*-test	Adjusted *P* value
	*n*	M	SD	n	M	SD		
Medication access	793	0.61	19.68	1392	0.53	22.42	−3.60	.002
Medication delays	790	0.18	11.71	1386	0.15	13.55	−2.38	.09
Coverage for tests or treatments	781	0.20	12.04	1321	0.19	14.98	−0.39	1.00
Experienced step therapy and poor health outcomes	142	0.85	9.51	254	0.84	10.47	−0.26	1.00
Financial barriers delay medication	914	0.17	12.03	1622	0.17	15.71	0.15	1.00
Use of copay programs	813	0.59	19.65	1460	0.54	22.95	−2.40	.12

	White, non-Hispanic^c^			Non-White			*t*-test	Adjusted *P* value
	*n*	M	SD	n	M	SD		
Medication access	1731	0.55	22.29	222	0.63	11.42	2.12	.21
Medication delays	1755	0.16	15.46	227	0.25	7.45	3.10	.01
Coverage for tests or treatments	1692	0.19	16.42	218	0.25	7.33	1.98	.24
Experienced step therapy and poor health outcomes	299	0.85	9.01	68	0.87	7.18	0.47	1.00
Financial barriers delay medication	2000	0.18	17.30	259	0.25	7.89	2.47	.08
Use of copay programs	1940	0.55	23.44	252	0.56	11.46	0.28	1.00

	Lives in an area with concentrated poverty^d^			Lives in an area without concentrated poverty			*t*-test	Adjusted *P* value
	*n*	M	SD	n	M	SD		
Medication access	137	0.56	8.64	1758	0.56	21.96	−0.16	1.00
Medication delays	^e^							
Coverage for tests or treatments	^e^							
Experienced step therapy and poor health outcomes	34	0.97	5.54	322	0.84	8.23	−3.64	.001
Financial barriers delay medication	170	0.22	6.04	2327	0.16	17.74	−1.81	.42
Use of copay programs	143	0.57	8.91	1961	0.54	23.09	−0.71	1.00

	≥65 years			≤64 years			*t*-test	Adjusted *P* value
	*n*	M	SD	n	M	SD		
Medication access	461	0.34	12.14	1839	0.62	24.88	11.22	.000
Medication delays	473	0.06	5.49	1816	0.19	17.13	8.55	<.0001
Coverage for tests or treatments	435	0.09	6.12	1782	0.23	18.34	8.27	<.0001
Experienced step therapy and poor health outcomes					^e^			
Financial barriers delay medication	656	0.14	9.40	2341	0.17	18.48	1.87	.37
Use of copay programs	534	0.30	12.37	1890	0.61	25.13	13.52	<.0001

	Employer or union health insurance only			Medicare only			*t*-test	Adjusted *P* value
	*n*	M	SD	n	M	SD		
Medication access	1197	0.60	19.33	230	0.43	9.59	−5.00	.000
Medication delays	1211	0.18	13.58	235	0.09	4.55	−4.08	.000
Coverage for tests or treatments	1180	0.23	14.82	220	0.09	4.44	−5.95	<.0001
Experienced step therapy and poor health outcomes					^e^			
Financial barriers delay medication	1339	0.17	13.91	298	0.22	8.02	2.19	.17
Use of copay programs	1305	0.64	20.00	291	0.34	9.69	−9.75	<.0001

	1 + Financial barriers and trade-offs^f^			No financial barriers and trade-offs			*t*-test	Adjusted *P* value
	*n*	M	SD	n	M	SD		
Advanced specialty medication	936	0.80	22.67	1285	0.72	23.53	−4.48	<.0001
Academic setting	943	0.38	17.52	1378	0.35	19.51	−1.91	.06
White, non-Hispanic	887	0.85	22.59	1295	0.91	24.08	4.47	<.0001
Lives in area with concentrated poverty	867	0.09	8.68	1263	0.06	8.30	−2.89	.004
65 years and older	1024	0.11	10.47	1456	0.29	18.75	11.57	<.0001
Employer or union health insurance	626	0.86	19.97	958	0.80	21.67	−3.22	.001

^a^Advanced specialty medication is defined as the patient takes a biologic therapy, biosimilar, or targeted synthetic small molecule medication.

^b^Academic setting is defined as the patient receives IBD healthcare in an academic setting.

^c^White, non-Hispanic is defined as the patient identifies as White, non-Hispanic.

^d^Area of concentrated poverty is defined as areas with a poverty rate of 20% or more (based on the Census Bureau’s benchmark definition of “high poverty area.”).

^e^This test did not meet one or more of the reporting criteria.

^f^1+ Financial barriers and trade-offs is defined as the patient, respondent, or someone else in the household made financial sacrifices to pay for healthcare or insurance costs related to their IBD including borrowing money from friends or family; taking out any type of loan; seeking out the aid of a charity or nonprofit organization; increasing credit card debt; cutting back on food, clothing, or basic household items; putting off vacations or major household purchases; taking money out of retirement, college, or other long-term saving account; taking an extra job or working more hours; changing living situation; using up all or most of savings; and/or using internet or social media to raise funds to pay for medical care.

Abbreviation: M, mean.

#### Medication delays

Among respondents who prescribed medication for their IBD in the prior 12 months, 12.2% (*n* = 251) reported not being able to get all their IBD prescription medication(s). Additionally, 16.8% (*n = *341) experienced a significant delay of one or more months waiting for insurance to approve their IBD prescription. Consequently, many experienced adverse health events (eg, increased pain, a new flare, etc.; 69.4%, *n = *752) and effects on their daily life (unable to work, attend school, or do daily activities, such as cooking and caring for family; 48.7%, *n = *315) as a result. Respondents were more likely to experience a significant delay of one or more months waiting for insurance to approve their prescription if they were prescribed an advanced specialty medication (M* *= 0.19, SD = 16.76, *t*(2287) = −7.56, *P* < .0001), identified as something other than White, non-Hispanic (M = 0.25, SD = 7.45, *t*(1981) = 3.10, *P* = .01), were 64 years and younger (M = 0.19, SD = 17.13, *t*(2288) = 8.55, *P* < .0001), or had employer or union only health insurance (M = 0.18, SD = 13.58, *t*(1445) = −4.08, *P* < .001) when compared to their analysis counterpart (Table 4).

#### Coverage for tests or treatments

In the prior 12 months, 88.5% (*n = *2015) of respondents needed at least one test or treatment for their IBD care, such as blood and stool tests, endoscopic procedures, radiology scans, diagnostic imaging, surgery, or treatment for complications. Of these respondents, 19.9% (*n = *391) experienced difficulties with insurance covering their test or treatments, that is they “never” or only “sometimes” were able to get insurance to cover them. Respondents were more likely to experience coverage difficulties if on an advanced specialty medication (M* *= 0.21, SD = 16.88, *t*(2042) = −2.77, *P* = .03), 64 years and younger (M = 0.23, SD = 18.34, *t*(2216) = 8.27, *P* < .0001), or had employer or union only health insurance (M = 0.23, SD = 14.82, *t*(1399) = −5.95, *P* < .0001) when compared to their analysis counterpart (Table 4).

#### Respondent confidence in navigating insurance barriers

**Figure 2.**
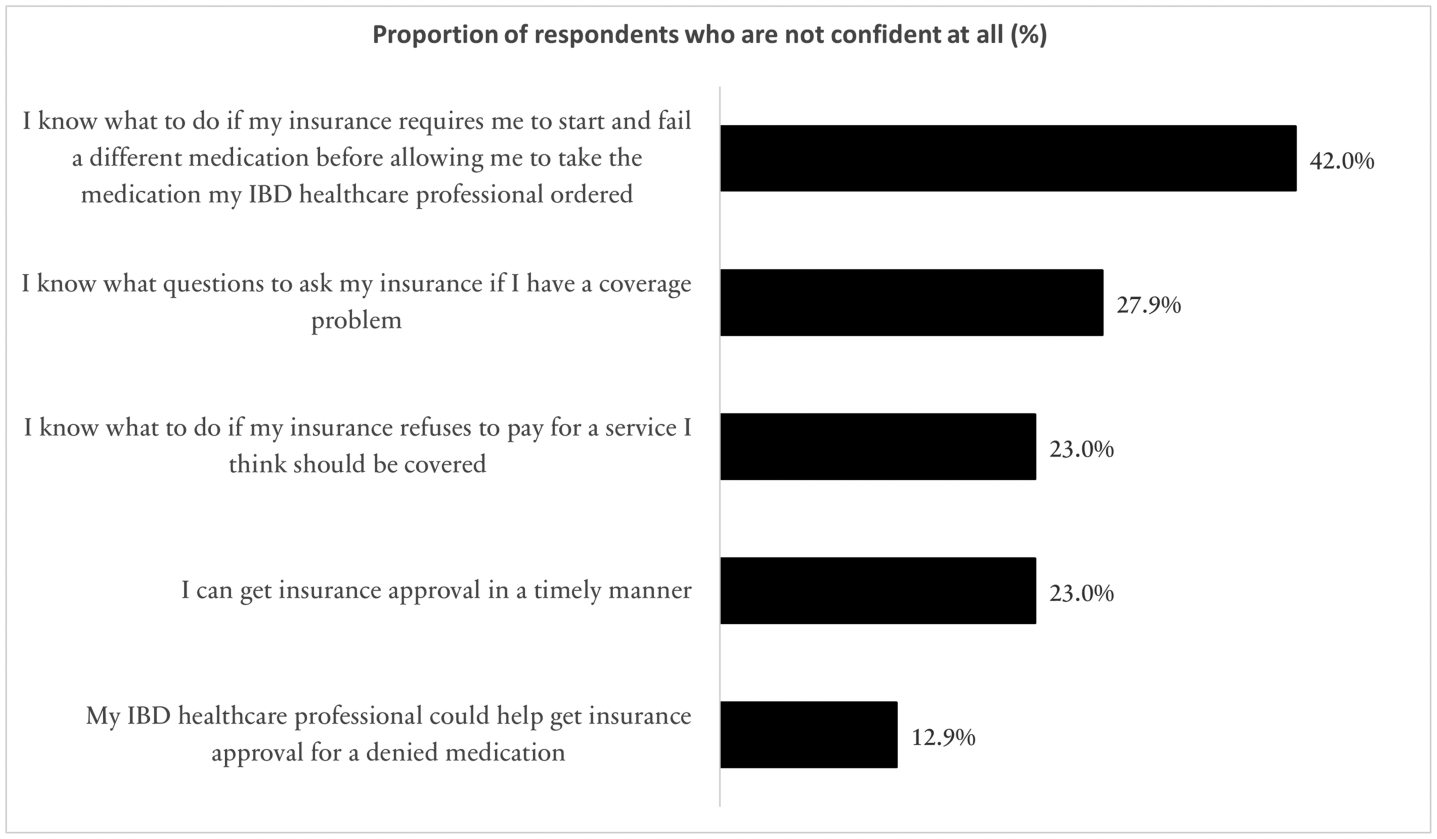
Confidence in navigating health insurance barriers.

In terms of respondent confidence in navigating insurance barriers, 42% (*n* = 919) were “not confident at all” in knowing what to do if their insurance required step therapy prior to the healthcare professional’s originally requested medication (Figure 2). Approximately 1 out of 4 respondents, were not confident at all in knowing what questions to ask their insurance if faced with coverage problems, knowing what to do if insurance refuses to pay for a service that should be covered, or that they could get insurance approval in a timely manner. One out of 10 respondents were not confident at all that their IBD healthcare professional could help obtain insurance approval for denied treatment.

### Step Therapy Mandates

At the time of the survey, 34 states had passed Step Therapy legislation; 80% (*n* = 1826) of respondents reported that they lived in one of those states. Of respondents who were prescribed IBD medications in the prior 12 months, 24.8% (*n = *480) experienced step therapy mandates (insurance required that they try a different medication before the preferred medication prescribed by their IBD healthcare professional). Of those who experienced step therapy mandates, 85.4% (*n = *321) experienced adverse health outcomes when unable to get or delayed in getting their prescribed IBD medications. When we examined differences by groups, patients were more likely to experience step therapy mandates and have poor health outcomes if they lived in an area with concentrated poverty (M = 0.97, SD = 5.54) compared to respondents who lived in an area without concentrated poverty (M = 0.84, SD = 8.23), *t*(355) = −3.64, *P* = .001 (Table 4).

### Financial Barriers to Care

**Figure 3.**
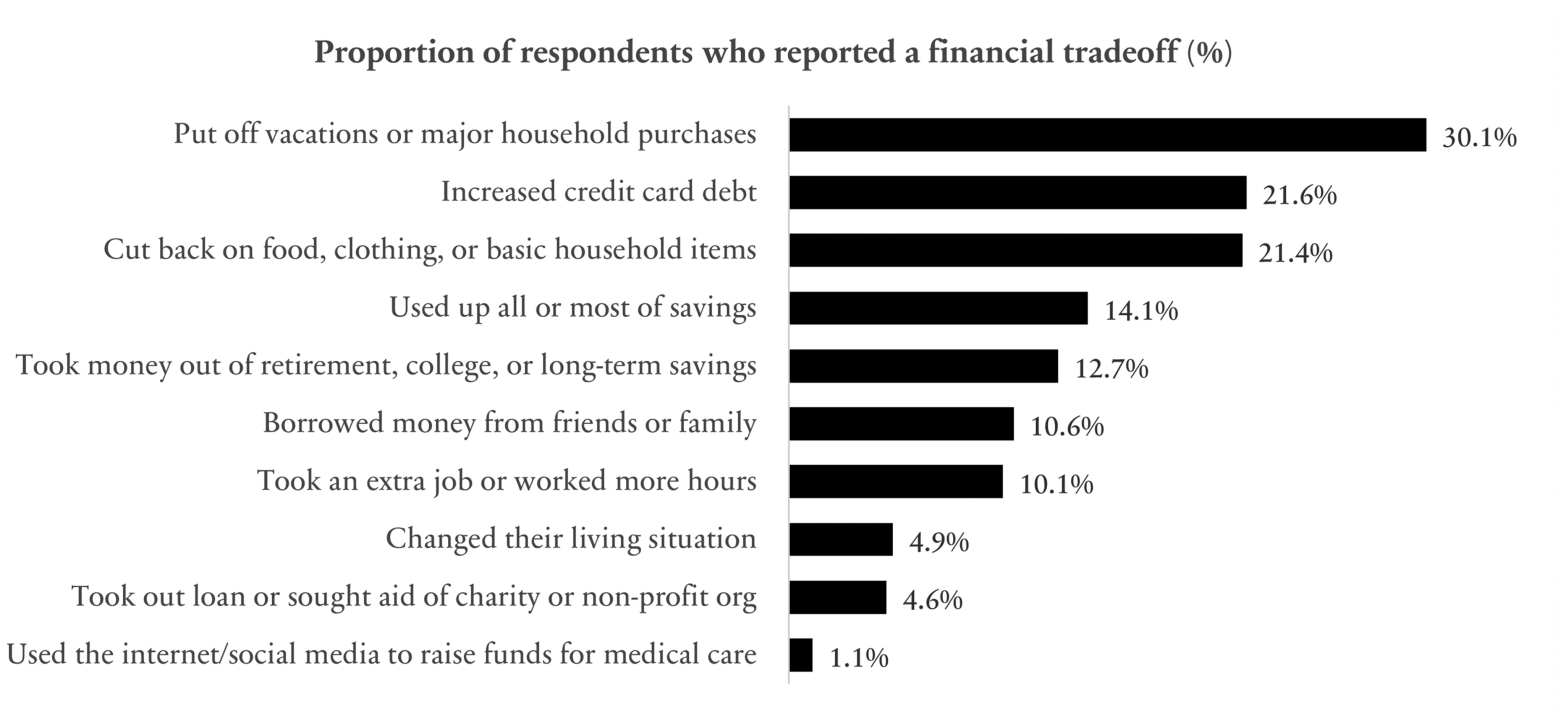
Financial barriers to IBD healthcare. Proportion of respondents who indicated yes that “In order to pay your healthcare or insurance costs related to your IBD, have you or someone else in the household done any of the following in the past 12 months?” to one or more of the items. Respondents could check all that apply.

A quarter of respondents (24.4%, *n = *556) reported having trouble paying or were unable to pay IBD-related medical bills, with 41.1% (*n = *922) experiencing one or more financial barriers or trade-offs (eg, increased credit card debt, put off vacations or major household purchases [see variable definitions in Supplementary Table S1]) in the prior 12 months (Figure 3). Respondents were more likely to experience one or more financial barriers or trade-offs (versus those who reported none) if the patient used an advanced specialty medication (M* *= 0.80, SD = 22.67, *t*(2220) = −4.48, *P* < .0001), or lived in an area with concentrated poverty (M = 0.09, SD = 8.68, *t*(2129) = −2.89, *P* = .004). Respondents were more likely to report no financial barriers or trade-offs (versus those who report one or more) if they identified as White, non-Hispanic (M = 0.85, SD = 22.59, *t*(2181) = 4.47, *P* < .0001), were 65 years and older (M = 0.11, SD = 10.47, *t*(2479) = 11.57, *P* < .0001), or had Medicare only health insurance (M = 0.80, SD = 21.67, *t*(1583) = −3.22, *P* < .0001; Table 4).

### Financial Barriers to Obtaining Medications

More than 3 out of 5 respondents (62.7%, *n = *438) experienced financial barriers to obtaining their prescribed medications (eg, did not get, delayed, used an alternative cheaper medication, or did not take their IBD medication as prescribed due to the cost). Consequently, a significant number of respondents experienced adverse health events (66.1%, *n = *396) and effects on their daily life (51.2%, *n = *206) as a result. Respondents were more likely to report that financial barriers interfered with their ability to get medication if they were not on an advanced specialty medication (M* *= 0.25, SD = 11.67), compared to those who did take an advanced specialty medication (M* *= 0.16, SD = 15.93, *t*(2359) = 4.15, *P* < .001).

### Use of Copay Programs

More than half of respondents (54.2%, *n = *1232) utilized a copay program in the prior 12 months with almost 1 out of 5 (17.1%, *n = 211*) reporting using up or running out of copay assistance funds. Respondents were more likely to report using a copay program if on an advanced specialty medication (M* *= 0.70, SD = 23.54, *t*(2174) = −21.18, *P* < .0001), were 64 years and younger (M = 0.61, SD = 25.13, *t*(2423) = 13.52, *P* < .0001), or had employer or union only health insurance (M = 0.64, SD = 20.00, *t*(1595) = −9.75, *P* < .0001) when compared to their analysis counterpart (Table 4). Additionally, respondents who reported experiencing one or more financial barriers or trade-offs (M = 0.64, SD = 21.64) were also more likely to use a copay program when compared to those who did not experience financial barriers or trade-offs (M = 0.48, SD = 22.06*, t*(2387) = −7.93, *P* < .0001).

## Discussion

The first Foundation assessment of healthcare access was published in 2017. It identified that within the United States, many patients with IBD endorsed healthcare-related financial worry, leading to deferment of necessary medical care.^2^ Subsequent publications have highlighted that associated IBD-related healthcare expenditure continues to rise^25,26^; however, IBD management has since shifted to incorporate the use of(1) advanced therapies earlier in the disease course and (2) intensive monitoring and therapeutic targets, in an effort to obtain remission sooner, limit IBD-related complications, and minimize hospitalizations and surgeries.^27,28^ Despite these advances over the past 7 years, our updated assessment highlights that, similar to the 2017 survey findings, patients with IBD continue to experience significant financial burdens and access barriers within the US healthcare system, due to insurance restrictions, step therapy mandates, and unaffordable cost of care. This remains a significant concern as such barriers hinder patients from timely and vital access to care, including effective medications and various tests necessary to assess and achieve optimal disease outcomes. As evidenced by our survey, 62.7% of respondents reported experiencing treatment delays or deferment due to IBD medication-associated costs, which resulted in patients experiencing adverse health effects (66.1%) and negative effects on daily living (51.2%).

The associated cost of IBD has been projected to be as high as $42 000 annually, with advanced therapies being one of the biggest expenditures.^29^ As a result, several cost-saving measures, such as prior authorizations and step therapy, have been implemented by insurance companies. These interventions are often confusing and frustrating due to the lack of standardization and transparency with the processes, which requires additional resources and time to partake in completing these steps.^6,30^ Several studies have highlighted that these processes are associated with treatment delays (up to 34 days) and can lead to worsening disease in the interim.^31-33^ Simultaneously, prior studies have also identified a disconnect between insurance policies and current guideline recommendations, such as requiring stepwise drug failure before approval of an advanced therapy requested by the healthcare professional and not covering drug-level testing despite clinical utility.^11,34-36^ Our survey findings reiterate that these barriers still exist in IBD care, especially for those on advanced therapies. Moreover, this effect seems more pronounced in academic settings, as there is often a higher volume of patients, who are more likely to have commercial and non-Medicare insurance and severe or complicated diseases warranting immediate diagnostic and treatment care.

At the time of this survey, 37 states had passed step therapy reform legislation. However, these state laws apply only to state-regulated plans with the majority of patients being enrolled in federally regulated plans. Even with the protections of step therapy reform legislation, patients continue to experience challenges accessing their medication due to step therapy. Among the respondents who experienced step therapy mandates in the prior 12 months, all resided in one of the 37 states that have passed step therapy reform legislation. This does not suggest that reform legislation is not effective and instead suggests that it is essential to pass a federal law so that all patients would be protected from unreasonable step therapy requirements. Our analysis was not able to fully explore the strength of existing step therapy mandates. Future surveys should explore the effectiveness of step therapy reform legislation and evaluate its impact on patient’s access to medication.

Most survey respondents reported they see a healthcare professional within a gastroenterologist office for IBD care; however, given the growing prevalence and incidence of IBD, non-IBD specialists, including primary care and general gastroenterologists, are caring for these patients as well.^37^ Future surveys should encompass patients receiving IBD care outside of IBD specialists to further understand these patients’ experiences with accessing healthcare and to ascertain if there are differences in outcomes based on the type of healthcare professional providing IBD care.

Treatment delays were more pronounced in patients less than 65 years old, possibly due to commercial plans generally requiring prior authorizations for all advanced IBD therapies, whereas Medicare covers infusion therapies for all FDA-approved indications under Part B without prior authorization.^38^ Interestingly, prior studies identified an opposite trend, noting a longer length of time to prior authorization approval with government-related plans; however, these studies evaluated both Medicare and Medicaid, whereas our study only analyzed Medicare coverage. The inclusion of more insurance plans should be a focus area in the future.^32,39^ In addition, our survey did not delineate between self-administered versus infusible advanced therapies, and this should be an area of interest for future surveys, considering the differences between Medicare Part B and Part D coverage.

One positive finding was respondents are taking advantage of assistance programs; respondents taking advanced therapies and those less than 65 years old reported utilizing copay programs. These findings are consistent with practice, as most copay programs are available for specialty medications and for patients with commercial insurance. Of note, respondents who reported experiencing one or more financial barriers or trade-offs were more likely to use a copay program when compared to those who did not experience financial barriers or trade-offs. Future studies should focus on exploring this association further.

Although not directly assessed in our survey, it is worth noting that most patients with IBD who are faced with an insurance issue felt hopeless and incapable of navigating the insurance issue, thus eventually giving up.^40^ Unfortunately, the lack of direct, timely, and covered access to IBD-related care remains a significant concern given its impact on disease outcomes and overall patients’ quality of life.

Another theme suggested by our survey was the prevalence of racial disparities. In the 2017 survey by Rubin et al.,^2^ several demographic factors such as age, sex, education, and insurance type, but not race, were associated with increased risk for healthcare-related financial worry, access to insurance, and delayed medical care. However, in our study, respondents who did not self-identify as White and non-Hispanic were more likely to experience increased financial barriers and delays with medications. In a systematic review and meta-analysis involving 58 studies, Black and Hispanic patients were found to have poorer access to IBD specialists, suboptimal symptom control, and lower quality of life.^33^ This is concerning because the incidence of IBD in non-White populations has quickly increased with more recent data suggesting that this population may have a higher incidence of more severe disease.^4,41,42^ There are also pertinent cultural factors in the non-White population that affect IBD management such as potential distrust in physicians and concerns about long-term medication use and medication-associated harms.^41,42^ Race may also be a factor to consider when deciding on IBD therapeutic options as we advance into an era of precision medicine.^43^ Thus, racial disparities represent another set of barriers to optimal IBD management and healthcare access and should also be a focus area for reform.

The Foundation is dedicated to advocating for patients with IBD and offers several resources to both healthcare professionals and patients/caregivers to help optimize IBD access and care. There is a webpage dedicated to managing the cost of IBD and contains information on how to identify the best insurance plan, navigate denials and copay accumulators, and sign up for patient assistance programs (https://www.crohnscolitisfoundation.org/patientsandcaregivers/managing-the-cost-of-ibd). On the Foundation’s webpage for healthcare professionals, appeal letter templates for both medications and tests/procedures are provided to help reduce administrative burdens (https://www.crohnscolitisfoundation.org/science-and-professionals/program-materials/appeal-letters). The Foundation is also partaking in advocacy efforts relating to step therapy, including passing legislative reform designed to enact patient protections against this requirement imposed by payers (https://www.crohnscolitisfoundation.org/get-involved/be-an-advocate/steptherapy). Lastly, the Foundation is committed to diversity, equity, and inclusion efforts to help address health inequities that many marginalized communities face (https://www.crohnscolitisfoundation.org/about/diversity). Based on the survey results the Foundation will update and revise existing resources and collaborate with other organizations to address the gaps identified by the survey. The Foundation will leverage the survey to inform the implementation of new advocacy and diversity, equity, and inclusion initiatives in collaboration with other coalitions and organizations. Lastly, where needed, the Foundation will seek support to launch additional research initiatives to ensure the data needed to address the gaps identified in the survey are addressed.

### Limitations and Generalizability

The strengths of this study include the large number of respondents, including patients and caregivers, and high inclusion of many patients on advanced therapy, especially considering the treatment practice shift to now utilize these types of treatments earlier in the disease course. Conversely, while we determined the survey to be the best study methodology for mass distribution to capture various perspectives and experiences, and although we made concerted efforts to reach diverse populations, including through partnering with external organizations, our population was not as diverse as hoped. Given that the United States lacks a unified health system or central health database, data about the prevalence of IBD among the US population is limited and difficult to determine. However, we recognize that our survey sample was not representative of the entire IBD population in the United States. A recent study estimated that the prevalence of IBD is nearly twice as high among non-Hispanic White Americans compared to Black, Hispanic, and Asian Americans^3^; our survey sample was predominantly White, non-Hispanic respondents. In addition, survey respondents tended to have a longer diagnosis of IBD, health insurance, and a high level of education. While the apparent lack of representation does limit the external validity of the study, one could hypothesize that the populations not robustly captured (eg, Black, Asian, Hispanic, no insurance, and other SDOH risks) are likely to have even greater difficulty receiving a diagnosis, accessing healthcare, medications, and treatments, and increased financial barriers. The literature highlights that underrepresented and vulnerable IBD patients often face additional disparities in accessing healthcare and obtaining a diagnosis.^44-48^

Moreover, while our study offers valuable insights into the relationships between age, insurance status, and barriers to care, we recognize that we did not control for the correlations among key SDOH, specifically age and insurance. A chi-square analysis indicates a significant relationship between these categorical variables, X2 (2, *N* = 1612) = 1078.61, *P* < .0001, which may have influenced our findings. These factors, along with other demographic and socioeconomic characteristics, are crucial contributors to disparities in healthcare access and utilization. The complex, multidimensional nature of SDOH highlights the need for careful identification and analysis of patterns within the data. To achieve a more comprehensive understanding, future research should employ multivariate methods to delve deeper into these associations, providing a detailed analysis of how age, insurance, and other SDOH intersect to impact healthcare access and outcomes.

Inherent to the survey methodology, duplicate responses may have been captured due to anonymous entry and reliance on self-reported data, which may not be fully accurate depending on how respondents defined certain terms, such as step therapy and what qualifies as pharmaceutical assistant programs. Additionally, medical nutrition in Crohn’s disease is a growing area of interest, as exclusive enteral nutrition has been shown to improve symptoms and induce remission.^49^ Due to insufficient response rates analysis could not be completed to evaluate whether medical nutrition was impacted by coverage; however, this will be a focus for future surveys.

Future surveys should also focus on other evolving trends within healthcare, such as copay accumulators, maximizer plans, and increasing biosimilar availability, which are influencing IBD-related costs and access.^50^ In addition, future directions also include identifying how to increase response rates and diversity of respondents with future surveys and may be further guided by engaging with more communities and organizations that serve rural, urban, and diverse populations as well as focus groups with minority IBD patients.

## Conclusion

Despite the significant and numerous advancements within IBD, patients with IBD continue to experience challenges accessing healthcare, including medications, treatments, and healthcare professionals. Patients and their families experience significant healthcare costs and make trade-offs, such as taking less of their medication than prescribed or putting off major household purchases, to afford their healthcare costs. Ongoing awareness and advocacy efforts focused on healthcare system reform, achieving health equity, and policies to further minimize care disparities and barriers remain vital.

## Supplementary Data

Supplementary data is available at *Inflammatory Bowel Diseases* online.

izae237_suppl_Supplementary_Tables

izae237_suppl_Supplementary_Data

## References

[1] Park KT , Ehrlich OG, Allen JI, et al The cost of inflammatory bowel disease: an initiative from the Crohn’s & Colitis Foundation. Inflamm Bowel Dis. 2020;26(1):1-10. doi: https://doi.org/10.1093/ibd/izz104 31112238 PMC7534391

[2] Rubin DT , Feld LD, Goeppinger SR, et al The Crohn’s and Colitis Foundation of America survey of inflammatory bowel disease patient health care access. Inflamm Bowel Dis. 2017;23(2):224-232. doi: https://doi.org/10.1097/MIB.0000000000000994 27997434

[3] Lewis JD , Parlett LE, Funk MLJ, et al Incidence, prevalence, and racial and ethnic distribution of inflammatory bowel disease in the United States. Gastroenterology. 2023;165(5):1197-1205.e2. doi: https://doi.org/10.1053/j.gastro.2023.07.003 37481117 PMC10592313

[4] Liu JJ , Abraham BP, Adamson P, et al The current state of care for black and hispanic inflammatory bowel disease patients. Inflamm Bowel Dis. 2023;29(2):297-307. doi: https://doi.org/10.1093/ibd/izac124 35816130 PMC10210746

[5] Regueiro MD , McAnallen SE, Greer JB, Perkins SE, Ramalingam S, Szigethy E. The inflammatory bowel disease specialty medical home: a new model of patient-centered care. Inflamm Bowel Dis. 2016;22(8):1971-1980. doi: https://doi.org/10.1097/MIB.0000000000000819 27135486

[6] Spencer EA , Abbasi S, Kayal M. Barriers to optimizing inflammatory bowel disease care in the United States. Therap Adv Gastroenterol. 2023;16:17562848231169652. doi: https://doi.org/10.1177/17562848231169652. Accessed January 7, 2024. https://journals.sagepub.com/doi/10.1177/17562848231169652?url_ver=Z39.88-2003&rfr_id=ori:rid:crossref.org&rfr_dat=cr_pub%20%200pubmed PMC10164253 37163167

[7] Anyane-Yeboa A , Quezada S, Rubin DT, Balzora S. The impact of the social determinants of health on disparities in inflammatory bowel disease. Clin Gastroenterol Hepatol. 2022;20(11):2427-2434. doi: https://doi.org/10.1016/j.cgh.2022.03.011 35307597

[8] Barnes EL , Loftus EV, Kappelman MD. Effects of race and ethnicity on diagnosis and management of inflammatory bowel diseases. Gastroenterology. 2021;160(3):677-689. doi: https://doi.org/10.1053/j.gastro.2020.08.064 33098884

[9] Nguyen NH , Khera R, Ohno-Machado L, Sandborn WJ, Singh S. Prevalence and effects of food insecurity and social support on financial toxicity in and healthcare use by patients with inflammatory bowel diseases. Clin Gastroenterol Hepatol. 2021;19(7):1377-1386.e5. doi: https://doi.org/10.1016/j.cgh.2020.05.056 32526341 PMC7987215

[10] Najeeb H , Yasmin F, Surani S. Emerging role of biosimilars in the clinical care of inflammatory bowel disease patients. World J Clin Cases. 2022;10(14):4327-4333. doi: https://doi.org/10.12998/wjcc.v10.i14.4327 35663066 PMC9125297

[11] Anderson KL , Anand R, Feuerstein JD. Insurance companies’ poor adherence to guidelines for moderate-to-severe ulcerative colitis/crohn’s disease management. Am J Gastroenterol. 2024;119(7):1417-1420. doi: https://doi.org/10.14309/ajg.0000000000002720 38417043

[12] Step Therapy Reform. Crohn’s & Colitis Foundation. Accessed April 14, 2024. https://www.crohnscolitisfoundation.org/get-involved/be-an-advocate/steptherapy

[13] Click B , Regueiro M. The inflammatory bowel disease medical home: from patients to populations. Inflamm Bowel Dis. 2019;25(12):1881-1885. doi: https://doi.org/10.1093/ibd/izz062 30934057

[14] Regueiro M , Click B, Anderson A, et al Reduced unplanned care and disease activity and increased quality of life after patient enrollment in an inflammatory bowel disease medical home. Clin Gastroenterol Hepatol. 2018;16(11):1777-1785. doi: https://doi.org/10.1016/j.cgh.2018.04.007 29654918 PMC6185823

[15] Regueiro M. Medical homes for patients with inflammatory bowel disease. Gastroenterol Hepatol. 2017;13(6):375-377. PMC5495046 28690455

[16] National Center for Health Statistics. National Health Interview Survey, 2021. Public-use data file and documentation. 2022. Accessed August 1, 2022. https://www.cdc.gov/nchs/nhis/data-questionnaires-documentation.htm

[17] Centers for Medicare & Medicaid Services. Medicare advantage and prescription drug plan CAHPS® survey, Baltimore, MD. Accessed June 28, 2022. www.MA-PDPCAHPS.org

[18] Centers for Medicare & Medicaid Services. Hospital consumer assessment of healthcare providers and systems (HCAHPS) survey. Accessed June 28, 2022. https://www.hcahpsonline.org

[19] Kaiser Family Foundation. Kaiser Family Foundation/LA Times Survey of adults with employer-sponsored health insurance. 2019. Accessed August 30, 2022. https://files.kff.org/attachment/Report-KFF-LA-Times-Survey-of-Adults-with-Employer-Sponsored-Health-Insurance

[20] U.S. Census Bureau, Changes in Areas with Concentrated Poverty: 2000-2010. American Community Survey Briefs ACS-27 (PDF). U.S. Government Printing Office; 2014. Accessed October 9, 2014.

[21] U.S. Census Bureau, Areas with Concentrated Poverty: 2006-2010. American Community Survey Briefs ACSBR/10-17 (PDF). U.S. Government Printing Office; 2011.

[22] U.S. Census Bureau, American Community Survey 2018-2022. Accessed November 1, 2022. https://data.census.gov/

[23] SAS Institute Inc. SAS/STAT® 15.2 User’s Guide. Cary, NC: SAS Institute Inc; 2024.

[24] von Elm E , Altman DG, Egger M, Pocock SJ, Gøtzsche PC, Vandenbroucke JP; STROBE Initiative. The Strengthening the Reporting of Observational Studies in Epidemiology (STROBE) statement: guidelines for reporting observational studies. Lancet. 2007;370(9596):1453-1457. doi: https://doi.org/10.1016/S0140-6736(07)61602-X 18064739

[25] NHSR 152: health care utilization among U.S. adults with inflammatory bowel disease, 2015–2016. Centers for Disease Control and Prevention; 2021. doi: https://doi.org/10.15620/cdc:100471 33663650

[26] Singh S , Qian AS, Nguyen NH, et al Trends in U.S. health care spending on inflammatory bowel diseases, 1996-2016. Inflamm Bowel Dis. 2022;28(3):364-372. doi: https://doi.org/10.1093/ibd/izab074 33988697 PMC8889287

[27] Berg DR , Colombel JF, Ungaro R. The role of early biologic therapy in inflammatory bowel disease. Inflamm Bowel Dis. 2019;25(12):1896-1905. doi: https://doi.org/10.1093/ibd/izz059 30934053 PMC7185690

[28] Turner D , Ricciuto A, Lewis A, et al; International Organization for the Study of IBD. STRIDE-II: an update on the Selecting Therapeutic Targets in Inflammatory Bowel Disease (STRIDE) Initiative of the International Organization for the Study of IBD (IOIBD): determining therapeutic goals for treat-to-target strategies in IBD. Gastroenterology. 2021;160(5):1570-1583. doi: https://doi.org/10.1053/j.gastro.2020.12.031 33359090

[29] Kahn-Boesel O , Cautha S, Ufere NN, Ananthakrishnan AN, Kochar B. A narrative review of financial burden, distress, and toxicity of inflammatory bowel diseases in the United States. Am J Gastroenterol. 2023;118(9):1545-1553. doi: https://doi.org/10.14309/ajg.0000000000002345 37224301

[30] Bhat S , Zahorian T, Robert R, Farraye FA. Advocating for patients with inflammatory bowel disease: how to navigate the prior authorization process. Inflamm Bowel Dis. 2019;25(10):1621-1628. doi: https://doi.org/10.1093/ibd/izz013 30753551

[31] Choi DK , Cohen NA, Choden T, Cohen RD, Rubin DT. Delays in therapy associated with current prior authorization process for the treatment of inflammatory bowel disease. Inflamm Bowel Dis. 2023;29(10):1658-1661. doi: https://doi.org/10.1093/ibd/izad012 36715294

[32] Loeb L , Nasir A, Picco MF, Hashash JG, Kinnucan JA, Farraye FA. Prior authorization of biologics in the management of inflammatory bowel disease. Inflamm Bowel Dis. 2023;29(9):e37. doi: https://doi.org/10.1093/ibd/izad088 37196096

[33] Shah NB , Zuckerman AD, Hosteng KR, et al Insurance approval delay of biologic therapy dose escalation associated with disease activity in patients with inflammatory bowel disease. Dig Dis Sci. 2023;68(12):4331-4338. doi: https://doi.org/10.1007/s10620-023-08098-7 37725192

[34] Sofia MA , Feuerstein JD, Narramore L, Chachu KA, Streett S. White Paper AGA: American Gastroenterological Association Position Statement: the future of IBD care in the United States-removing barriers and embracing opportunities. Clin Gastroenterol Hepatol. 2024;22(5):944-955. doi: https://doi.org/10.1016/j.cgh.2024.01.050 38428707

[35] Yadav A , Foromera J, Feuerstein I, Falchuk KR, Feuerstein JD. Variations in health insurance policies regarding biologic therapy use in inflammatory bowel disease. Inflamm Bowel Dis. 2017;23(6):853-857. doi: https://doi.org/10.1097/MIB.0000000000001153 28509816

[36] Yadav A , Vasquez P, Dolgin NH, Falchuk KR, Feuerstein JD. Variations in insurance policies regarding adherence to the AGA guideline for therapeutic drug monitoring in IBD. J Clin Gastroenterol. 2019;53(6):e239-e242. doi: https://doi.org/10.1097/MCG.0000000000001144 30439759

[37] Bennett AL , Munkholm P, Andrews JM. Tools for primary care management of inflammatory bowel disease: do they exist? World J Gastroenterol. 2015;21(15):4457-4465. doi: https://doi.org/10.3748/wjg.v21.i15.4457 25914455 PMC4402293

[38] Whitmire N , Schlueter M, Kirkpatrick M. Advanced therapies for inflammatory bowel disease: navigating payor and financial challenges. Curr Gastroenterol Rep. 2024;26(3):68-76. doi: https://doi.org/10.1007/s11894-024-00916-w 38243152 PMC10937800

[39] Rao V , Dharia I, Gibilisco J, et al Delay in prior authorization of biologic therapy: another possible cause of healthcare disparity in IBD patients. J Natl Med Assoc. 2024;116(1):13-15. doi: https://doi.org/10.1016/j.jnma.2023.09.009 38036315

[40] Philippou A , Birhanu B, Biello A, Keefer L, Gorbenko K. A mixed-methods assessment of the impact of insurance issues on the emotional and physical health of patients with inflammatory bowel disease. Inflamm Bowel Dis. 2022;28(12):1851-1858. doi: https://doi.org/10.1093/ibd/izac022 35191977

[41] Odufalu FD , Aboubakr A, Anyane-Yeboa A. Inflammatory bowel disease in underserved populations: lessons for practice. Curr Opin Gastroenterol. 2022;38(4):321-327. doi: https://doi.org/10.1097/MOG.0000000000000855 35762691 PMC10332404

[42] Shah S , Shillington AC, Kabagambe EK, et al Racial and ethnic disparities in patients with inflammatory bowel disease: an online survey. Inflamm Bowel Dis. 2024;30(9):1467-1474. doi: https://doi.org/10.1093/ibd/izad194 37703380 PMC11369073

[43] Greywoode R , Petralia F, Ullman TA, Frederic Colombel J, Ungaro RC. Racial difference in efficacy of golimumab in ulcerative colitis. Inflamm Bowel Dis. 2023;29(6):843-849. doi: https://doi.org/10.1093/ibd/izac161 35913121 PMC10233400

[44] Damas OM , Kuftinec G, Khakoo NS, et al Social barriers influence inflammatory bowel disease (IBD) outcomes and disproportionally affect Hispanics and non-Hispanic Blacks with IBD. Ther Adv Gastroenter. 2022;15:1-14. doi: https://doi.org/10.1177/17562848221079162 PMC8958706 35356362

[45] Walker C , Allamneni C, Orr J, et al Socioeconomic status and race are both independently associated with increased hospitalization rate among Crohn’s disease patients. Sci Rep. 2018;8(1):1-6. 29507339 10.1038/s41598-018-22429-z PMC5838155

[46] Vest BM , Kulak JA, Homish GG. Caring for veterans in US Civilian Primary Care: qualitative interviews with primary care providers. Fam Pract. 2019;36(3):343-350. doi: https://doi.org/10.1093/fampra/cmy078 30281097 PMC6531893

[47] Call KT , McAlpine DD, Garcia CM, et al Barriers to care in an ethnically diverse publicly insured population: is health care reform enough? Med Care. 2014;52(8):720-727. doi: https://doi.org/10.1097/MLR.0000000000000172 25023917

[48] Lazar M , Davenport L. Barriers to health care access for low income families: a review of literature. J Community Health Nurs. 2018;35(1):28-37. doi: https://doi.org/10.1080/07370016.2018.1404832 29323941

[49] Comeche JM , Caballero P, Gutierrez-Hervas A, et al Enteral nutrition in patients with inflammatory bowel disease. Systematic review, meta-analysis, and meta-regression. Nutrients. 2019;11(11):2657. doi: https://doi.org/10.3390/nu11112657 31689999 PMC6893586

[50] Angyal A , Bhat S. Biosimilars in IBD: what every clinician needs to know. Curr Gastroenterol Rep. 2024;26(3):77-85. doi: https://doi.org/10.1007/s11894-023-00913-5 38243154

