# Supplementary material for: Healthcare Access for Patients With Inflammatory Bowel Disease in the United States: A Survey by the Crohn’s & Colitis Foundation
Source: Inflamm Bowel Dis. 2024 Oct 8;31(7):1819–32. doi: 10.1093/ibd/izae237 (PMC12235136; doi:10.1093/ibd/izae237)
Supplement: izae237_suppl_Supplementary_Tables [file izae237_suppl_supplementary_tables.docx]

**Supplement Tables**

**Table S1. Variable definitions**

| **Variable** | **Respondent population/ question logic** | **Definition** |
| --- | --- | --- |
| Disease severity | All respondents | Respondent describes his/her IBD over the past 6 months:   - Severe: Constantly active, giving symptoms every day OR often active, giving symptoms most days - Moderate: Sometimes active, giving symptoms on some days OR occasionally active, giving symptoms 1-2 days a month - Mild: Rarely active, giving symptoms only a few days in past 6 months OR was well in past 6 months, what he/she considers remission or absence of symptoms |
| Poverty | All respondents | To create a categorization of poverty, the respondents’ self-reported home ZIP Code was matched to US Census American Community Survey data to determine the percentage of the population that lives in poverty. These were grouped into four poverty rate level categories and then combined into “not a poverty area” and “poverty area” using thresholds developed by the US Census Bureau. |
| Adverse health outcomes | All respondents | In prior 12 months when did not take IBD medication as prescribed, experienced at least one of the following:   - Decreased quality of life (e.g., decreased appetite, disturbed sleep, negative impact on mental health) - Increased pain - Took a nonsteroid medication to treat symptoms (e.g., anti-diarrheal medication, such as Imodium/loperamide or pain medication, such as Norco, Tylenol, Tramadol) - Took steroid/corticosteroid to treat symptoms (e.g., Prednisone) - A new flare - Worsening of an existing flare - An abscess, fistula, or infection - Emergency department visit - Hospitalization - Surgery to treat their IBD |
| **Definitions for experiencing difficulties related to care for IBD** | | |
| **Medication Access**  Experienced problems accessing their IBD prescriptions due to health insurance | Respondents who in the prior 12 months were prescribed any medication for their IBD | Problems accessing IBD prescriptions due to health insurance defined as experiencing at least one of the following in the prior 12 months:   - Insurance would not cover a medication prescribed by their IBD healthcare professional - Insurance would not cover the medication dose or frequency prescribed by their IBD healthcare professional - Had to wait more than 2 days to fill a prescription because the pharmacy needed an additional approval from their insurance - Insurance required that they try a different medication before the preferred medication prescribed by their IBD healthcare professional - Spent more than 2 hours on the phone with their insurance provider trying to get a prescription covered |
| **Medication delays**  Experienced insurance approval delays of one month or longer for their IBD prescriptions | Respondents who in the prior 12 months were prescribed any medication for their IBD | In prior 12 months, the longest delay they experienced waiting for insurance to approve their prescription medications to treat their IBD was 1 month or longer or they never received their medications |

**Table S1 Continued**

| **Coverage for tests and treatments**  Insurance approval or coverage delays for tests or treatments | All respondents | In prior 12 months, at least once needed one of the following tests or treatments, but insurance never or sometimes covered the test or treatment,   - Therapeutic drug monitoring test - Calprotectin stool test - Other tests (blood and stool tests; endoscopic procedures, such as colonoscopy, upper endoscopy, sigmoidoscopy, capsule endoscopy; radiology scans; and diagnostic imaging, such as X-rays, upper GI scans, CT scans, MRE) - Other treatments (Surgery, such as colectomy, proctocolectomy; treatment for abscesses and fistulas, such as antibiotics, surgery; and treatment for extraintestinal complications of IBD, e.g, iron infusions for anemia, supplements for osteoporosis) |
| --- | --- | --- |
| **Step therapy mandates**  Experienced step therapy mandates | Respondents who in the prior 12 months were prescribed any medication for their IBD | In prior 12 months, insurance required that patient try a different medication before the preferred medication prescribed by their IBD healthcare professional |
| Experienced **financial barriers or tradeoffs** | All respondents | In order to pay their healthcare or insurance costs related to their IBD, patient or someone else in the family did any of the following:   - Borrowed money from friends or family - Took out any type of loan (e.g., an additional mortgage) - Sought the aid of a charity or non-profit organization - Increased credit card debt - Cut back on food, clothing, or basic household items - Put off vacations or major household purchases - Took money out of retirement, college, or other long-term savings account - Took an extra job or worked more hours - Changed living situation (e.g., moved in with friends or family) - Used up all or most of savings - Used internet or social media to raise funds to pay for medical care |
| **Financial barriers interfered with ability to obtain medications** | All respondents | In prior 12 months, any of the following was true for patient regarding their IBD medication:   - Did not get medication because of cost - Skipped medication doses to save money - Took less medication to save money - Delayed filling a prescription to save money - Asked doctor for a lower cost medication to save money - Brought medication from another country to save money - Used alternative therapies to save money |
| **Used a copay program** | All respondents | Received a discount on a medication, either through a coupon, copay card, drug company patient assistance program, or some other kind of medication discount |

**Table S2. Population definitions and analysis comparison groups**

| **Variable** | **Group 1** | **Group 2** |
| --- | --- | --- |
| Advanced specialty medications | Yes  Among patients prescribed IBD medication in prior 12 months, those who **have taken** a biologic therapy, biosimilar, or targeted synthetic small molecule for their IBD | No  Among patients prescribed IBD medication in prior 12 months, those who **have not taken** **or do not know if they have taken** a biologic therapy, biosimilar, or targeted synthetic small molecule for their IBD |
| Academic setting | Yes  The patient typically receives their care at a Healthcare professional’s office, clinic, OR VA Medical Center or VA Outpatient clinic that is also an academic setting (i.e., affiliated with a university, college, or equivalent) | No  The patient does not or is unsure if their care at a Healthcare professional’s office, clinic, OR VA Medical Center or VA Outpatient clinic that is also an academic setting (i.e., affiliated with a university, college, or equivalent). |
| White, non-Hispanic | Identifies as White, non-Hispanic | Do not identify as White, non-Hispanic |
| Poverty | Yes  Patient lives in a ZIP code that has concentrated poverty. Meaning the poverty rates are 20.0% or more. | No  Patient lives in a zip code that does not have concentrated poverty. Meaning the poverty rates are 19.9% or less. |
| Age | 64 years or younger | 65 years or older |
| Health insurance | Has employer or union-based health insurance only | Has Medicare only |
