# Supplementary material for: Healthcare Access for Patients With Inflammatory Bowel Disease in the United States: A Survey by the Crohn’s & Colitis Foundation
Source: Inflamm Bowel Dis. 2024 Oct 8;31(7):1819–32. doi: 10.1093/ibd/izae237 (PMC12235136; doi:10.1093/ibd/izae237)
Supplement: izae237_suppl_Supplementary_Data [file izae237_suppl_supplementary_data.docx]

**Supplement 1. Survey Instruments**

**Inflammatory Bowel Disease Health Access Survey Adults with IBD**

ABOUT YOU

Before we start the survey, we would like to know some information about you.

**A1.**  Have you ever been told by a doctor or other healthcare professional that you had any of the following diseases or conditions?

Mark all that apply

1 □ Celiac disease

2 □ Crohn’s disease

3 □ Eosinophilic esophagitis

4 □ Gastroesophageal reflux disease (Reflux)

5 □ Gastroparesis

6 □ Inflammatory bowel disease unclassified (IBD-U), includes indeterminate colitis

7 □ Irritable bowel syndrome (IBS)

8 □ Ulcerative colitis

9 🔾 I have not been diagnosed with any of these conditions

| **Programmer Box** |
| --- |
| - HARD CHECK (Response required) - IF A1 = 2, 6, or 8, GO TO A3. ONE IBD DIAGNOSIS selected - If A1 = 2, 6, AND/or 8, GO TO A2. two or more IBD diagnosIs selected - IF A1 DOES NOT EQUAL 2, 6, or 8, go to a4. No IBD DIAGNOSIS selected, go to Caregiver screening |

**A2.** Which IBD condition were you **most recently diagnosed** with? If you are unsure, select the diagnosis that you receive the most treatment for.

1 🔾 Crohn’s disease

2 🔾 Ulcerative colitis

3 🔾 Inflammatory bowel disease unclassified (IBD-U), includes indeterminate colitis

4 🔾 My diagnosis changes frequently between Crohn’s disease, ulcerative colitis, indeterminate colitis, and/or IBD-U

| **Programmer Box** |
| --- |
| - HARD CHECK (Response required) |

**A3.** Do you live in the United States or a United States territory?

1 🔾 Yes

2 🔾 No

3 🔾 I prefer not to answer

| **Programmer Box** |
| --- |
| - hARD CHECK - IF A3 = 1, GO TO A3A. passed LIVING IN THE US screening. - If A3 = 2 OR 3, go to *SCREEN OUT- LIVING IN THE US*. failed LIVING IN THE US screening, RESPONDENTS ARE SCREENED OUT. |

**A3a.** Which state or territory do you live in?

**________** [DROPDOWN]

| **Programmer Box** |
| --- |
| - hARD CHECK - IF A3A = ALABAMA OR NEBRASKA, GO TO A4A. - IF A3A = MISSISSIPPI OR PUERTO RICO, GO TO A4B - ELSE GO TO A4C. |

**A4A.** What is your age?

**________** [DROPDOWN]

95 🔾 Prefer not to disclose

96 🔾 Don’t know

| **Programmer Box** |
| --- |
| - HARD CHECK - dropdown RANGE IS 17 YEARS OR YOUNGER -110 - IF A4A = 19 - 110, GO TO ACCESS TO HEALTHCARE PROFESSIONALS passed Patient age screening. - IF A4a = 17 YEARS OR YOUNGER – 18 YEARS, GO TO *SCREEN OUT – AGE* PAGE. FAILED PATIENT AGE SCREENING, RESPONDENTS ARE SCREENED OUT. - If A4a = 95 or 96, GO TO *SCREEN OUT – AGE* PAGE. FAILED PATIENT AGE SCREENING, RESPONDENTS ARE SCREENED OUT. |

**A4B.** What is your age?

**________** [DROPDOWN]

95 🔾 Prefer not to disclose

96 🔾 Don’t know

| **Programmer Box** |
| --- |
| - HARD CHECK - dropdown RANGE IS 17 YEARS OR YOUNGER -110 - IF A4b = 21 - 110, GO TO ACCESS TO HEALTHCARE PROFESSIONALS passed Patient age screening. - IF A4b = 17 YEARS OR YOUNGER – 20 YEARS, GO TO *SCREEN OUT – AGE* PAGE. FAILED PATIENT AGE SCREENING, RESPONDENTS ARE SCREENED OUT. - If a4b = 95 or 96, GO TO *SCREEN OUT – AGE* PAGE. FAILED PATIENT AGE SCREENING, RESPONDENTS ARE SCREENED OUT. |

**A4C.** What is your age?

**________** [DROPDOWN]

95 🔾 Prefer not to disclose

96 🔾 Don’t know

| **Programmer Box** |
| --- |
| - HARD CHECK - dropdown RANGE IS 17 YEARS OR YOUNGER -110 - IF A4c = 18 - 110, GO TO ACCESS TO HEALTHCARE PROFESSIONALS passed Patient age screening. - IF A4c = 17 YEARS OR YOUNGER, GO TO *SCREEN OUT – AGE* PAGE. FAILED PATIENT AGE SCREENING, RESPONDENTS ARE SCREENED OUT. - If A4c = 95 or 96, GO TO *SCREEN OUT – AGE* PAGE. FAILED PATIENT AGE SCREENING, RESPONDENTS ARE SCREENED OUT. |

ACCESS TO HEALTHCARE PROFESSIONALS

This survey is about your experiences with healthcare for your Crohn’s disease, ulcerative colitis, or inflammatory bowel disease unclassified (IBD-U). We will refer to these conditions as inflammatory bowel disease (IBD) throughout the survey. Please only answer the questions from your experiences with your IBD condition and exclude any other health conditions that are unrelated to your IBD.

The next questions are about who provides and where you usually receive your IBD care.

**B1.**  Think about the healthcare professional who you usually see for your IBD care. This person is the primary person you work with to make health decisions about your IBD.

What type of **healthcare professional** do you **usually** go to for your IBD care?

1 🔾 Gastroenterologist (MD/DO)

2 🔾 Nurse practitioner/Physician’s assistant in a gastroenterologist’s office

3 🔾 Primary care physician/General practitioner (MD/DO)

4 🔾 Nurse practitioner/Physician’s assistant in a primary care physician/General practitioner’s office

5 🔾 Other healthcare professional

6 🔾 Prefer not to disclose

7 🔾 Not applicable – I do not have a usual healthcare professional

**B2.**  Think about the **place** you go to for your IBD healthcare visits with your healthcare professional. If you receive infusions at a different place, do not consider that location in your answer.

Is there a **place** you **usually** go to receive your IBD care?

1 🔾 Yes, there is a place I usually go to receive care, and I receive majority of my care on site

2 🔾 Yes, there is a place I usually go to receive care, but I receive the majority of my care through telehealth visits

3 🔾 No, there is not a place I usually go to receive care

GO TO B3

4 🔾 Don’t know

| **Programmer Box** |
| --- |
| - IF B2 = 1 or 2, go to b2a. - If B2 = 3 or 4, go to b4. |

**B2a.**  Where do you **usually go** to receive your IBD care? If you primarily use virtual telehealth visits, think about the place your healthcare professional is located.

1 🔾 Healthcare professional’s office or clinic

2 🔾 VA Medical Center or VA Outpatient clinic

3 🔾 Public health clinic, community health center, or tribal clinic

GO TO B3

4 🔾 Holistic or alternative medicine provider

5 🔾 Hospital emergency room

6 🔾 Urgent care clinic

7 🔾 Retail clinic (e.g., CVS or Walgreens)

8 🔾 Some other place

| **Programmer Box** |
| --- |
| - IF B2a = 1 or 2, go to b2B. - If B2a = 3, 4, 5, 6, 7 or 8, go to b3. |

**B2b.** Some healthcare professionals practice in academic medical centers which provide graduate medical training and research. Academic medical centers are affiliated with medical schools.

Is this practice or clinic an **academic setting** (i.e., affiliated with a university, college, or equivalent)?

1 🔾 Yes

2 🔾 No

3 🔾 Don’t know

**B3.** Which of the following resources are available **at the** **practice or clinic** you **usually go** to receive your IBD care?

MARK ONLY ONE PER ROW

|  | AVAILABLE | NOT AVAILABLE | DON’T KNOW |
| --- | --- | --- | --- |
| a. Emotional support (e.g., psychologist or social worker who provides short-term therapy, group therapy, or patient support groups) | 1 🔾 | 2 🔾 | 3 🔾 |
| b. IBD surgical specialists (e.g., Colorectal surgeon) | 1 🔾 | 2 🔾 | 3 🔾 |
| c. Nutritionist/dietitian | 1 🔾 | 2 🔾 | 3 🔾 |
| d. Ostomy clinic | 1 🔾 | 2 🔾 | 3 🔾 |
| e. Pharmacist to help with IBD medication | 1 🔾 | 2 🔾 | 3 🔾 |
| f. Patient liaison or other staff who can help navigate insurance (e.g., prior authorizations), prescription renewals, and other paperwork | 1 🔾 | 2 🔾 | 3 🔾 |
| g. Alternative medicine (e.g., Pilates, acupuncture, reiki, or yoga to help with stress management) | 1 🔾 | 2 🔾 | 3 🔾 |
| h. Clinical trials | 1 🔾 | 2 🔾 | 3 🔾 |

**B4.** In the last 12 months, **when you needed care right away**, how often did you get care as soon as you needed?

1 🔾 Never

2 🔾 Sometimes

3 🔾 Usually

4 🔾 Always

5 🔾 Not applicable – I did not need care right away in the past 12 months

**B5.** There are many reasons people delay getting medical care. During the past 12 months, did you **delay or not get medical care** for your IBD for any of the following reasons?

MARK ONLY ONE PER ROW

|  | YES | NO | NOT APPLICABLE OR I DO NOT HAVE INSURANCE |
| --- | --- | --- | --- |
| a. Difficulty finding a doctor, healthcare professional, clinic, or hospital that accepts my insurance | 1 🔾 | 2 🔾 | 3 🔾 |
| b. An appointment wasn’t available when I needed it | 1 🔾 | 2 🔾 | 3 🔾 |
| c. Couldn’t get to the doctor’s office or clinic when it was open | 1 🔾 | 2 🔾 | 3 🔾 |
| d. It takes too long to get to the doctor’s office or clinic | 1 🔾 | 2 🔾 | 3 🔾 |
| e. Too busy with work or other commitments to take the time | 1 🔾 | 2 🔾 | 3 🔾 |
| f. Couldn’t get through to the office or clinic on the telephone | 1 🔾 | 2 🔾 | 3 🔾 |
| g. Didn’t have transportation | 1 🔾 | 2 🔾 | 3 🔾 |
| h. Waiting for insurance approval | 1 🔾 | 2 🔾 | 3 🔾 |
| i. Other (*specify*) ___________________________________________ | 1 🔾 | 2 🔾 | 3 🔾 |

MEDICATIONS, TESTS, AND TREATMENTS

The next questions are about medications for your IBD. When answering these questions, think about the medications prescribed by your IBD healthcare professional. Diet, nutrient rich formula, and exclusive enteral nutrition (EEN) are not considered medications in this section.

**C1.** Think about the medications you take to manage your IBD. Please consider both new and renewed prescriptions that are a part of your regular treatment or that you take occasionally to treat your symptoms.

In the last 12 months, were you prescribed **any medication** for your IBD?

1 🔾 Yes

2 🔾 No

GO TO C8

3 🔾 Don’t know

| **Programmer Box** |
| --- |
| - IF c1 = 1, go to C2. - If c1 = 2 OR 3, go to c8. |

**C2.** Corticosteroids suppress the immune system. Corticosteroids include medications such as Prednisone (Deltasone®), Prednisolone (Oraped®, Prelone®, Pediapred®), Methylprednisolone (A-Methapred®, Depo-Medrol®, Medrol Dosepak®, Solu-Medrol®), and Budesonide (Entocort® EC, UCERIS™).

In the last 12 months, have you taken **corticosteroids** for your IBD?

1 🔾 Yes

2 🔾 No

GO TO C3

3 🔾 Don’t know

| **Programmer Box** |
| --- |
| - IF C2 = 1, go to C2a. - If c2 = 2 OR 3, go to c3. |

**C2a.** In the last 12 months, what is the **longest amount of time** you took corticosteroids **daily** for your IBD?

1 🔾 I did not take corticosteroids daily

2 🔾 Every day for less than 2 weeks

3 🔾 Every day for 2 to 4 weeks

4 🔾 Every day for more than 4 weeks, but less than 12 weeks

5 🔾 Every day for 12 weeks or more

**C3.** Biologic therapies are bioengineered drugs that target very specific molecules involved in the inflammatory process. Biosimilars are similar, near identical copies of already approved biologic therapies. Biologic therapies and biosimilars are given as an infusion or an injection.

Examples of biologic therapies and biosimilars include Adalimumab [including biosimilars] (Humira®, Cyltezo™, Hyrimoz™, Abrilada™, Amjevita®, HADLIMA, Hulio®), Certolizumab pegol (Cimzia®), Golimumab (Simponi®) and Infliximab [including biosimilars and unbranded] (Remicade®, Renflexis®, Avsola™, Inflectra™, IXIFI™), Natalizumab (Tysabri®), Risankizumab (Skyrizi®), Ustekinumab (Stelara®), and Vedolizumab (Entyvio®).

Targeted synthetic small molecules help reduce inflammation by specifically targeting parts of the immune system that play a role in inflammation. They are taken orally in pill form. There are three targeted synthetic small molecules approved to treat ulcerative colitis – Ozanimod (Zeposia®), Tofacitinib (Xeljanz®), Upadacitinib (RINVOQ®).

In the last 12 months, have you taken a **biologic therapy, biosimilar, or targeted synthetic small molecule** for your IBD?

1 🔾 Yes

2 🔾 No

3 🔾 Don’t know

**C4.** Sometimes patients have difficulties obtaining their IBD prescription medications. In the past 12 months, did you experience any of the following issues?

MARK ONLY ONE PER ROW

|  | YES | NO | NOT APPLICABLE OR I DO NOT HAVE INSURANCE |
| --- | --- | --- | --- |
| a. Insurance would not cover a medication prescribed by my IBD healthcare professional | 1 🔾 | 2🔾 | 3 🔾 |
| b. Insurance would not cover the medication dose or frequency prescribed by my IBD healthcare professional | 1 🔾 | 2🔾 | 3 🔾 |
| c. Had to wait more than 2 days to fill a prescription because the pharmacy needed an additional approval from my insurance | 1 🔾 | 2🔾 | 3 🔾 |
| d. Insurance requires that I try a different medication before the preferred medication prescribed by my IBD healthcare professional | 1 🔾 | 2🔾 | 3 🔾 |
| e. Spent more than 2 hours on the phone with my insurance provider trying to get a prescription covered | 1 🔾 | 2🔾 | 3 🔾 |
| f. Could not receive an infusion at my preferred location | 1 🔾 | 2🔾 | 3 🔾 |
| g. Had to take time off from work to be available to receive medication supplies at home | 1 🔾 | 2🔾 | 3 🔾 |
| h. Sought out a second opinion or made an appointment with another healthcare professional to assist with a denied insurance appeal | 1 🔾 | 2🔾 | 3 🔾 |
| i. Experienced clerical errors (e.g., prescription sent to wrong pharmacy, incorrect dosage provided, pharmacy could not find script, etc.) | 1 🔾 | 2🔾 | 3 🔾 |
| j. Decided not to fill a prescription because of possible side effects | 1 🔾 | 2🔾 | 3 🔾 |

**C5.** Think about the medications you take to manage your IBD. Please consider both new and renewed prescriptions that are a part of your regular treatment and medication that you take occasionally to treat your symptoms.

In the last 12 months, were you able to get **all** of your IBD prescription medication?

1 🔾 Yes GO TO C6

2 🔾 No

3 🔾 Don’t know

| **Programmer Box** |
| --- |
| - IF c5 = 1, go to c6. - If c5 = 0 OR D, go to C5a. |

**C5a.** In the last 12 months, how often did you get the **prescription** **medication** you needed to treat your IBD?

1 🔾 Never

2 🔾 Sometimes

3 🔾 Usually

4 🔾 Always

**C6.** In the past 12 months, what was the **longest delay** you experienced waiting for **insurance to approve** your prescription medications to treat your IBD?

1 🔾 Less than 1 week

2 🔾 1 week

3 🔾 2 to 3 weeks

4 🔾 1 to 3 months

5 🔾 4 or more months

6 🔾 I never received my medications

7 🔾 Not applicable – I did not experience a delay in the past 12 months

8 🔾 Not applicable – I do not have health insurance

**C6b.** In the last 12 months, when you were **unable** to get your IBD medication or when your IBD medication was **delayed**, did you experience any of the following?

Mark all that apply

1 □ Unable to work or attend school

2 □ Unable to do daily activities (e.g., cooking, caring for family)

3 □ Decreased quality of life (e.g., decreased appetite, disturbed sleep, negative impact on mental health)

4 □ Increased pain

5 □ Took a nonsteroid medication to treat symptoms (e.g., anti-diarrheal medication, such as Imodium/loperamide or pain medication, such as Norco, Tylenol, Tramadol)

6 □ Took steroid/corticosteroid to treat symptoms (e.g., Prednisone)

7 □ A new flare

8 □ Worsening of an existing flare

9 □ An abscess, fistula, or infection

10 □ Emergency department visit

11 □ Hospitalization

12 □ Surgery to treat my IBD

13 □ I did not experience any adverse events

14 □ Other *(specify)* _____________________________________________________________

NA 🔾 I was able to get all of my IBD medications and did not experience a delay

The next questions are about tests and treatments you need for your IBD care.

**C7.**  Therapeutic drug monitoring is testing that measures the amount of medication or the presence of anti-drug antibodies in your blood. This type of testing is often used when a patient is on biological therapies.

In the last 12 months, did you need **therapeutic drug monitoring**?

1 🔾 Yes

2 🔾 No

GO TO C8

3 🔾 Don’t know

| **Programmer Box** |
| --- |
| - IF c7 = 1, go to c7a. - If c7 = 2 OR 3, go to c8. |

**C7a.**  In the last 12 months, how often were you able to get **insurance to cover** your therapeutic drug monitoring?

If you have had more than one therapeutic drug monitoring test in the past 12 months, consider all and select the answer that best reflects your general experience.

1 🔾 Never

2 🔾 Sometimes

3 🔾 Usually

4 🔾 Always

5 🔾 Unsure – It is still under review by my insurance

6 🔾 Not applicable – I do not have health insurance

**C8.**  A calprotectin stool test is used to measure calprotectin - a protein in stool that indicates inflammation in the intestines. Healthcare professionals may use this test to monitor levels of inflammation and inform treatment.

In the last 12 months, did you need a **calprotectin stool test**?

1 🔾 Yes

2 🔾 No

GO TO C9

3🔾 Don’t know

| **Programmer Box** |
| --- |
| - IF c8 = 1, go to c8a. - If c8 = 2 OR 3, go to c9. |

**C8a.**  In the last 12 months, how often were you able to get **insurance to cover** your calprotectin stool test(s)?

If you have had more than one stool test in the past 12 months, consider all and select the answer that best reflects your general experience.

1 🔾 Never

2 🔾 Sometimes

3 🔾 Usually

4 🔾 Always

5 🔾 Unsure – It is still under review by my insurance

6 🔾 Not applicable – I do not have health insurance

**C9.**  In the last 12 months, did you need **other tests** for your IBD care?

Tests may include blood and stool tests, endoscopic procedures (e.g., colonoscopy, upper endoscopy, sigmoidoscopy, capsule endoscopy), radiology scans, and diagnostic imaging (e.g., X-rays, upper GI scans, CT scans, MRE).

1 🔾 Yes

2 🔾 No

GO TO C10

3 🔾 Don’t know

| **Programmer Box** |
| --- |
| - IF c9 = 1, go to C9a. - If c9 = 2 OR 3, go to c10. |

**C9a.** In the last 12 months, how often were you able to get **insurance to cover** your other test(s)?

If you have had more than one test in the past 12 months, consider all and select the answer that best reflects your general experience.

1 🔾 Never

2 🔾 Sometimes

3 🔾 Usually

4 🔾 Always

5 🔾 Unsure – It is still under review by my insurance

6 🔾 Not applicable – I do not have health insurance

**C10.**  In the last 12 months, did you need **other treatments** for your IBD care?

**Other treatments** may include surgery (e.g., colectomy, proctocolectomy), treatment for abscesses and fistulas (e.g., antibiotics, surgery), and treatment for extraintestinal complications of IBD (e.g., iron infusions for anemia, supplements for osteoporosis). Please exclude all IBD medications from your answer.

1 🔾 Yes

2 🔾 No

GO TO D1

3 🔾 Don’t know

| **Programmer Box** |
| --- |
| - IF c10 = 1, go to c10a. - If c10 = 2 or 3, go to d1. |

**C10a.** In the last 12 months, how often were you able to get **insurance to cover** your other treatment(s)?

If you have had more than one treatment in the past 12 months, consider all and select the answer that best reflects your general experience.

1 🔾 Never

2 🔾 Sometimes

3 🔾 Usually

4 🔾 Always

5 🔾 Unsure – It is still under review by my insurance

6 🔾 Not applicable – I do not have health insurance

FINANCIAL BARRIERS

The next set of questions are about paying for your IBD healthcare. This includes bills for healthcare professionals, hospitals, therapists, medication, equipment, and nursing home or home care.

**D1.** In the past 12 months, did you have trouble paying (e.g., late paying a bill, had to wait for your next paycheck) or were unable to pay for any medical bills related to your IBD care?

1 🔾 Yes

2 🔾 No

3 🔾 Don’t know

**D2.** In order to pay your healthcare or insurance costs related to your IBD, have you or someone else in the household done any of the following in the past 12 months?

MARK ONLY ONE PER ROW

|  | YES | NO | DON’T KNOW |
| --- | --- | --- | --- |
| a. Borrowed money from friends or family | 1 🔾 | 2🔾 | 3 🔾 |
| b. Took out any type of loan (e.g., an additional mortgage) | 1 🔾 | 2🔾 | 3 🔾 |
| c. Sought the aid of a charity or non-profit organization | 1 🔾 | 2🔾 | 3 🔾 |
| d. Increased credit card debt | 1 🔾 | 2🔾 | 3 🔾 |
| e. Cut back on food, clothing, or basic household items | 1 🔾 | 2🔾 | 3 🔾 |
| f. Put off vacations or major household purchases | 1 🔾 | 2🔾 | 3 🔾 |
| g. Took money out of retirement, college, or other long-term savings account | 1 🔾 | 2🔾 | 3 🔾 |
| h. Took an extra job or worked more hours | 1 🔾 | 2🔾 | 3 🔾 |
| i. Changed my living situation (e.g., moving in with family or friends) | 1 🔾 | 2🔾 | 3 🔾 |
| j. Used up all or most of my savings | 1 🔾 | 2🔾 | 3 🔾 |
| k. Used the internet or social media to raise funds to pay for medical care | 1 🔾 | 2🔾 | 3 🔾 |

**D3.** During the past 12 months, were any of the following true for you regarding your IBD medication?

Mark all that apply

1 □ I did not get my medication because of the cost

2 □ I skipped medication doses to save money

3 □ I took less medication to save money

4 □ I delayed filling a prescription to save money

5 □ I asked my doctor for a lower cost medication to save money

6 □ I bought medication from another country to save money

7 □ I used alternative therapies to save money

8 □ Other *(specify)*

NA 🔾 Cost did not interfere with my ability to get the medication

| **Programmer Box** |
| --- |
| - IF D3 = 1 – 8, go to D4. - If D3 = NA, go to D5. |

**D4.** During the past 12 months, when you **did not take your IBD medication as prescribed**, did you experience any of the following?

Mark all that apply

1 □ Unable to work or attend school

2 □ Unable to do daily activities (e.g., cooking, caring for family)

3 □ Decreased quality of life (e.g., decreased appetite, disturbed sleep, negative impact on mental health)

4 □ Increased pain

5 □ Took a nonsteroid medication to treat symptoms (e.g., anti-diarrheal medication, such as Imodium/loperamide or pain medication, such as Norco, Tylenol, Tramadol)

6 □ Took steroid/corticosteroid to treat symptoms (e.g., Prednisone)

7 □ A new flare

8 □ Worsening of an existing flare

9 □ An abscess, fistula, or infection

10 □ Emergency department visit

11 □ Hospitalization

12 □ Surgery to treat my IBD

13 □ Other *(specify)* _____________________________________________________________

NA 🔾 I did not experience any adverse events

**D5.** Coupons, copay cards, and copay assistance programs help patients with health insurance afford expensive prescription medications. Patient assistance programs offer free or low-cost prescription medications to people who do not have insurance or who have limited health insurance that covers only part of their healthcare expenses.

In the past 12 months, have you **received a discount on a medication**, either through a coupon, copay card, drug company patient assistance program, or some other kind of medication discount?

1 🔾 Yes

2 🔾 No

GO TO E1

3 🔾 Don’t know

| **Programmer Box** |
| --- |
| - IF D5 = 1, go to D5a. - If D5 = 2 OR 3, go to E1. |

**D5a.** Did you **use up or run out of** **one or more co-pay assistance(s)** before the end of the year (e.g., manufacturer payments were no longer applied to your deductible or out-of-pocket expenses)?

1 🔾 Yes

2 🔾 No

3 🔾 Don’t know

EXCLUSIVE ENTERAL NUTRITION (EEN)

| **Programmer Box** |
| --- |
| - IF A1 Only = 2 or a2 = 1, go to section e. Passed primary crohns disease screening. - IF a1 does not equal 2 OR A2 DOES NOT EQUAL 1, GO TO SECTION F. failed primary crohns disease screening. |

The next questions are about treating Crohn's disease with exclusive enteral nutrition (EEN), a nutrient rich formula used to provide all of a patient's nutrition. Patients drink the enteral formula or ingest it through a feeding tube (e.g., nasogastric (NG) tube, nasoduodenal (ND) tube, nasojejunal (NJ) tube, gastronomy tube (G-tube), or jejunostomy tube (J-tube)). Common formulas include Boost, Ensure, Orgain, Vital, Peptamen, and Modulen.

**E1.**  In the last 12 months, did your IBD healthcare professional prescribe exclusive enteral nutrition (EEN) or nutrient rich formula to treat your IBD?

1 🔾 Yes, through a tube (e.g., NG tube, ND tube, NJ tube, G-tube, or J-tube)

2 🔾 Yes, by mouth (e.g., oral or drink it)

3 🔾 No

GO TO F1

4 🔾 Don’t know

| **Programmer Box** |
| --- |
| - IF E1 = 1, 2, go to E2. - If B2 = 3 OR 4, go to F1. |

For the remainder of this section, we will refer to exclusive enteral nutrition (EEN) and nutrient rich formula as “formula”.

**E2.**  Does your insurance pay for your formula?

1 🔾 Yes, insurance covers all of the costs

2 🔾 Yes, but insurance only covers part of the costs

3 🔾 No, insurance does not cover any of the costs

4 🔾 Unsure – it is still under review by my insurance

5 🔾 Not applicable – I do not have health insurance

**E3.** In the last 12 months, were any of the following true for you regarding your formula?

Mark all that apply

1 □ I did not get my formula because of the cost

2 □ I skipped formula doses to save money

3 □ I took less formula to save money

4 □ I delayed filling a formula prescription to save money

5 □ I asked my doctor for a lower cost formula to save money

6 □ I bought formula from another country to save money

7 □ I tried a medication covered by insurance instead

8 □ I tried a different formula covered by my insurance instead of taking the one prescribed by my healthcare professional

9 □ I did not start formula

10 □ Other *(specify)*

NA 🔾 I had access to the prescribed formula and took it as instructed GO TO F1

| **Programmer Box** |
| --- |
| - IF E3 = 1, 2, 3, 4, 5, 6, 7, 8, 9, OR 10, go to E4. - If E3 = na, go to F1. |

**E4.**  During the time you could not get or did not take your formula as prescribed, did you experience any of the following with regards to your IBD?

Mark all that apply

1 □ Unable to work or attend school

2 □ Unable to do daily activities (e.g., cooking, caring for family)

3 □ Decreased quality of life (e.g., decreased appetite, disturbed sleep, negative impact on mental health)

4 □ Increased pain

5 □ Took a nonsteroid medication to treat symptoms (e.g., anti-diarrheal medication, such as Imodium/loperamide or pain medication, such as Norco, Tylenol, Tramadol)

6 □ Took steroid/corticosteroid to treat symptoms (e.g., Prednisone)

7 □ A new flare

8 □ Worsening of an existing flare

9 □ An abscess, fistula, or infection

10 □ Emergency department visit

11 □ Hospitalization

12 □ Surgery to treat my IBD

13 □ Other *(specify)* _____________________________________________________________

NA 🔾 I did not experience any adverse events

LEGISLATION AND ADVOCACY

| **Programmer Box** |
| --- |
| - IF A3a = one of the following states is selected: California, Oregon, Washington, Arizona, New Mexico, Colorado, South Dakota, Nebraska, Kansas, Oklahoma, Texas, Louisiana, Arkansas, Missouri, Iowa, Minnesota, Wisconsin, Illinois, Kentucky, Indiana, Tennessee, Mississippi, Ohio, Georgia, West Virginia, Virginia, North Carolina, Maryland, Delaware, Connecticut, Massachusetts, New York, pennsylvania, and Maine, go to section F. Passed step therapy state screening. - IF a3a = one of the following states is selected: Nevada, Utah, Wyoming, Montana, Vermont, New Hampshire, Idaho, North Dakota, South Carolina, Alabama, Florida, New Jersey, Alaska, Hawaii, Rhode Island, Michigan and DC, GO TO SECTION g. failed step therapy state screening. |

Earlier you indicated that you live in [FILL STATE FROM A3a]. The next question is about IBD legislation specific to this state.

**F1.** Step therapy, also known as “fail first,” is an insurance process which requires patients to try one or more medications, typically a generic or lower cost medicine, to treat a health condition. Patients must fail these medication(s) before allowing a “step up” to another medicine that may be more expensive for the insurer. **Your state has passed legislation** mandating changes to this insurance practice.

How familiar are you, if at all, with your state’s legislation to change step therapy or fail first protocols?

1 🔾 Very familiar

2 🔾 Somewhat familiar

3 🔾 Heard of, but know very little

4 🔾 Not at all familiar

AWARENESS OF FOUNDATION AND OTHER TOOLS

**G1.** Below is a list of resources used by people with IBD and their caregivers when they are having trouble getting medical care (e.g., getting insurance approval to see an IBD specialist or denial of a test or medication your IBD healthcare professional ordered).

Which of the following resources, if any, are you aware of or have used?

MARK ONLY ONE PER ROW

|  | NOT AWARE OF | AWARE OF | HAVE USED | NOT APPLICABLE OR I DO NOT HAVE INSURANCE |
| --- | --- | --- | --- | --- |
| a. IBD Insurance Checklist (i.e., a guide to evaluate and compare insurance plans) from the Crohn’s & Colitis Foundation | 1 🔾 | 2 🔾 | 3 🔾 | 4 🔾 |
| b. Tool to search patient financial assistance programs from the Crohn’s & Colitis Foundation | 1 🔾 | 2 🔾 | 3 🔾 | 4 🔾 |
| c. Customizable appeal letters (e.g., template letter if denied medication, or treatment) from the Crohn’s & Colitis Foundation | 1 🔾 | 2 🔾 | 3 🔾 | 4 🔾 |
| d. IBD Help Center from the Crohn’s & Colitis Foundation | 1 🔾 | 2 🔾 | 3 🔾 | 4 🔾 |
| e. In-person and online support groups | 1 🔾 | 2 🔾 | 3 🔾 | 4 🔾 |
| f. Resources from other patient advocacy or non-profit organizations | 1 🔾 | 2 🔾 | 3 🔾 | 4 🔾 |
| g. Resources from pharmaceutical companies (e.g., website and mailings with information about understanding insurance and financial assistance, nurse or videos to train patient in administering medication, and nurse ambassadors/hotlines) | 1 🔾 | 2 🔾 | 3 🔾 | 4 🔾 |
| h. Employee assistance programs (EAP) (e.g., an employee benefit that can include assessments, counseling, and referrals for additional services) | 1 🔾 | 2 🔾 | 3 🔾 | 4 🔾 |
| i. Other (*specify*) ____________________________________ | 1 🔾 | 2 🔾 | 3 🔾 | 4 🔾 |

**G2.** Think about the most recent time you had difficulties getting IBD care (e.g., insurance troubles, understanding medical bills, getting a healthcare appointment), did you reach out to family or friends for assistance?

1 🔾 Yes

2 🔾 No

3 🔾 Not applicable – I have not experienced any difficulties

**G3.**  Please indicate how confident **you** are in the following statements about your IBD care:

MARK ONLY ONE PER ROW

|  | NOT CONFIDENT AT ALL | SLIGHTLY CONFIDENT | FAIRLY CONFIDENT | VERY CONFIDENT | NOT APPLICABLE OR I DO NOT HAVE INSURANCE |
| --- | --- | --- | --- | --- | --- |
| a. I know what to do if my insurance refuses to pay for a service I think should be covered | 1 🔾 | 2 🔾 | 3 🔾 | 4 🔾 | 5 🔾 |
| b. I know what questions to ask my insurance if I have a coverage problem | 1 🔾 | 2 🔾 | 3 🔾 | 4 🔾 | 5 🔾 |
| c. I know what to do if my insurance requires me to start and fail a different medication before allowing me to take the medication my IBD healthcare professional ordered | 1 🔾 | 2 🔾 | 3 🔾 | 4 🔾 | 5 🔾 |
| d. My IBD healthcare professional could help get insurance approval for a denied medication | 1 🔾 | 2 🔾 | 3 🔾 | 4 🔾 | 5 🔾 |
| e. I can get insurance approval in a timely manner | 1 🔾 | 2 🔾 | 3 🔾 | 4 🔾 | 5 🔾 |

**G4.**  What other resources would be helpful when you are having trouble getting medical care, including medications?

Mark all that apply

1 □ Appeals process visual or graphic designed to show patients steps in the appeals process included expected timing and role of the patient

2 □ Suggested conversation starters to help patients feel more confident when discussing their care with their healthcare professional

3 □ Step-by-step brief videos describing what to do when denied coverage by my insurance company

4 □ A “Navigating Appeals” resource webpage for patients on the Crohn’s & Colitis Foundation website

5 □ Social sharing campaign for patients to share appeal successes

6 □ Other ideas (*specify*)

dk 🔾 Don’t know

YOUR EXPERIENCE

**H1.** Please use this space to share anything about your experiences accessing healthcare for your IBD that we have not asked about already and that it is important for us to know.

[Open ended textbox]

DEMOGRAPHIC CHARACTERISTICS AND CONTACT INFORMATION

The final set of questions ask about your background. Answers to these questions will be used to describe the type of people completing this survey.

**I1.** What year were you diagnosed with IBD? If you do not remember the year, please provide an estimate.

____________ [Dropdown]

| **Programmer Box** |
| --- |
| - drop down RANGE IS 2023 – … 2000; Before 2000; Don’t remember when I was diagnosed; prefer not to disclose |

**I2.** Which of the following best describe your IBD over the **past 6 months**?

1 🔾 Constantly active, giving me symptoms every day

2 🔾 Often active, giving me symptoms most days

3 🔾 Sometimes active, giving me symptoms on some days

4 🔾 Occasionally active, giving me symptoms 1-2 days a month

5 🔾 Rarely active, giving me symptoms only a few days in the past 6 months

6 🔾 I was well in the past 6 months, what I consider a remission or absence of symptoms

**I3.** Are you currently covered by any of the following types of health insurance or health coverage plans?

MARK ALL THAT APPLY

1 🔾 Not covered by health insurance

2 □ Employer or Union based insurance

3 □ Indian Health Service

4 □ Individual or Small Group Plan (e.g., through a state exchange)

5 □ Medicare

6 □ Medicaid, Medical Assistance, or any kind of non-Medicare government-assistance plan

7 □ Military health care (e.g., TRICARE, VA, CHAMP-VA)

8 □ Single service plan (e.g., dental, vision, prescriptions)

9 □ Other (*specify*) __________________________________________________________________

r 🔾 Prefer not to disclose

**I4.**  Are you of Hispanic, Latino/a, or Spanish origin?

1 🔾 Yes, Hispanic, Latino/a, or Spanish origin

2 🔾 No, not of Hispanic, Latino/a, or Spanish origin

3 🔾 Prefer not to disclose

**I5.** What is your race?

MARK ALL THAT APPLY

1 □ American Indian or Alaska Native

2 □ Asian

3 □ Black or African American

4 □ Native Hawaiian or Other Pacific Islander

5 □ White

6 □ Other self-identities (*specify*) ______________________________________________________________

r 🔾 Prefer not to disclose

**I6.** Do you identify as Middle Eastern or North African?

1 🔾 Yes

2 🔾 No

3 🔾 Prefer not to disclose

**I7.** What sex were you assigned at birth, on your original birth certificate?

1 🔾 Female

2 🔾 Male

3 🔾 Prefer not to disclose

**I8.** What is your current gender?

1 🔾 Female

2 🔾 Male

3 🔾 Transgender

0 🔾 I use a different term:

**I9.** What is the highest degree or level of school that you have completed?

1 🔾 Some school, but no high school diploma

2 🔾 High school diploma or GED

3 🔾 Some college credit, but no degree

4 🔾 Associate’s degree (e.g., AA, AS)

5 🔾 Bachelor’s degree (e.g., BA, BS)

6 🔾 Master’s degree or higher (e.g., MA, MS, PhD, MD, DO)

0 🔾 Other *(specify)* __________________________________________________________________

**I10.**  What is your current employment status?

MARK ALL THAT APPLY

1 □ Employed, full time

2 □ Employed, part time

3 □ Unemployed

4 □ Homemaker

5 □ Student

6 □ Retired

7 □ Unable to work

r 🔾 Prefer not to disclose

**I11.**  What is your home ZIP code?

| | | | | |

**I12.** Are you the **primary caregiver** of someone with IBD?

1 🔾 Yes

2 🔾 No GO TO END

| **Programmer Box** |
| --- |
| - IF I12 = 1, go to i12A. - If i12 = 2, go to THANK YOU. |

**I12a.** Would you like to take this survey again on behalf of a person you care for?

1 🔾 Yes, I would like to take the survey for another person in my care  GO TO C_A1 OF CAREGIVER SURVEY

2 🔾 No, I do not want to repeat the survey

GO TO END

3 🔾 No, I already completed the survey for all persons in my care

| **Programmer Box** |
| --- |
| - IF I12a = 1, go to A1 OF Caregiver survey. - If i12a = 2 or 3, go to THANK YOU. |

**Inflammatory Bowel Disease Health Access Survey – Caregivers of people with IBD**

ABOUT YOU

Before we start the survey, we would like to know some information about you.

**C_A1.**  Are you a **primary caregiver** for someone with IBD?

1 🔾 Yes GO TO C_A2

2 🔾 No GO TO END

3 🔾 Prefer not to answer

| **Programmer Box** |
| --- |
| - HARD CHECK - IF C_A1 = 1 or 2, GO TO C_A2. passed caregiver screening. - If C_A1 = d, go to *Screen OUT- FAMILIARITY WITH IBD CARE*. failed IBD screening, RESPONDENTS ARE SCREENED OUT. |

**C_A2.**  With which of the following IBD conditions was the person you care for **most recently diagnosed**?

If they have had multiple diagnoses, please mark the most recent diagnosis. If you are unsure, select the diagnosis that the person receives the most treatment for.

1 🔾 Crohn’s disease

2 🔾 Ulcerative colitis

3 🔾 Inflammatory bowel disease unclassified (IBD-U), includes indeterminate colitis

4 🔾 Their diagnosis changes frequently between Crohn’s disease, ulcerative colitis, indeterminate colitis, and/or IBD-U

5 🔾 Don’t know GO TO END

| **Programmer Box** |
| --- |
| - HARD CHECK - IF C_A2 = 1, 2, 3, 4, GO TO C_A3. passed IBD screening. - If C_A2 = d, go to *Screen OUT- FAMILIARITY WITH IBD CARE*. failed IBD screening, RESPONDENTS ARE SCREENED OUT. |

**C_A3.** What is the age of the person you care for?

__________ [Dropdown]

| **Programmer Box** |
| --- |
| - dropdown RANGE IS Infant (less than 1 years old), 1…-110; prefer not to disclose; don’t know - IF C_A3 = 0 – 110, go to C_A4. passed Patient age screening. - If C_A3 = r OR D, go to *Screen OUT- FAMILIARITY WITH IBD CARE*. failed patient age screening, RESPONDENTS ARE SCREENED OUT. |

**C_A4.**  How familiar are you with the person’s **current** IBD healthcare needs, care, and medications?

1 🔾 Not at all familiar

GO TO END

2 🔾 Slightly familiar

3 🔾 Somewhat familiar

4 🔾 Moderately familiar

5 🔾 Extremely familiar

| **Programmer Box** |
| --- |
| - IF C_A4 = 3, 4 or 5, GO TO C_A5. passed PATIENT HEALTH FAMILIARITY screening. - If C_A4 = 1 or 2, go to *Screen OUT- FAMILIARITY WITH IBD CARE*. failed CAREGIVER screening, RESPONDENTS ARE SCREENED OUT. |

**C_A5.**  Where does the person with IBD live?

__________ [Dropdown]

| **Programmer Box** |
| --- |
| - drop down RANGE IS all US states, Washington DC, and us territories; they live outside the united states; don’t know - IF C_A5 = any us state, washington dc or us territory. passed LIVING IN THE US screening. - If C_A5 = I live outside the United States OR D, go to *SCREEN OUT- LIVING IN THE US*. failed LIVING IN THE US screening, RESPONDENTS ARE SCREENED OUT. |

**C_A6.** What is **your** age?

1 🔾 Under 21 years old GO TO END

2 🔾 21 years or older

| **Programmer Box** |
| --- |
| - IF C_A6 = 2, GO TO C_b1. passed CAREGIVER age screening. - If C_A6 = 1, go to *screen out - age*. failed CAREGIVER age screening, RESPONDENTS ARE SCREENED OUT. |

ACCESS TO HEALTHCARE PROFESSIONALS

This survey is for caregivers of children or adults with Crohn’s disease, ulcerative colitis, or inflammatory bowel disease unclassified (IBD-U). We will refer to these conditions as inflammatory bowel disease (IBD) and the child or adult with IBD as “person with IBD” throughout the survey. Please only answer the questions about the person’s IBD healthcare experiences and exclude any other health conditions that are unrelated to their IBD.

If you are a caregiver for more than one person with IBD, answer the whole survey with one person in mind. You may repeat the survey for each additional person in your care.

The next questions are about who provides and where the person with IBD usually receive their IBD care.

**C_B1.** Think about the healthcare professional who the person with IBD usually sees for their IBD care. This person is the primary person they work with to make health decisions about their IBD.

What type of **healthcare professional** does the person with IBD **usually** go to for their IBD care?

1 🔾 Gastroenterologist (MD/DO)

2 🔾 Nurse practitioner/Physician’s assistant in a gastroenterologist’s office

3 🔾 Primary care physician/General practitioner (MD/DO)

4 🔾 Nurse practitioner/Physician’s assistant in a primary care physician/General practitioner’s office

5 🔾 Other healthcare professional

6 🔾 Prefer not to disclose

7 🔾 Not applicable – They do not have a usual healthcare professional

**C_B2.**  Think about the **place** the person with IBD goes to for their IBD healthcare visits with their healthcare professional. If they receive infusions at a different place, do not consider that location in your answer.

Is there a **place** the person with IBD **usually** goes to receive their IBD care?

1 🔾 Yes, there is a place they usually go to receive care, and they receive majority of their care on site

2 🔾 Yes, there is a place they usually go to receive care, but they receive the majority of their care through virtual telehealth visits

3 🔾 No, there is not a place they usually go to receive care

GO TO C_B4

4 🔾 Don’t know

| **Programmer Box** |
| --- |
| - IF B2 = 1, 2, go to b2a. - If B2 = 3 or 4, go to b4. |

**C_B2a.** Where does the person with IBD **usually go** to receive their IBD care? If they primarily use virtual telehealth visits, think about the place their healthcare professional is located.

1 🔾 Healthcare professional’s office or clinic

2 🔾 VA Medical Center or VA Outpatient clinic

3 🔾 Public health clinic, community health center, or tribal clinic

GO TO C_B3

4 🔾 Holistic or alternative medicine provider

5 🔾 Hospital emergency room

6 🔾 Urgent care clinic

7 🔾 Retail clinic (e.g., CVS or Walgreens)

8 🔾 Some other place

| **Programmer Box** |
| --- |
| - IF C_B2a = 1 or 2, go to C_b2B. - If C_B2a = 3, 4, 5, 6, 7 or 8, go to C_b3. |

**C_B2b.** Some healthcare professionals practice in academic medical centers which provide graduate medical training and research. Academic medical centers are affiliated with medical schools.

Is this practice or clinic an **academic setting** (i.e., affiliated with a university, college, or equivalent)?

1 🔾 Yes

2 🔾 No

3 🔾 Don’t know

**C_B3.** Which of the following resources are available **at the** **practice or clinic** the person with IBD **usually goes** to receive their IBD care?

MARK ONLY ONE PER ROW

|  | AVAILABLE | NOT AVAILABLE | DON’T KNOW |
| --- | --- | --- | --- |
| a. Emotional support (e.g., psychologist or social worker who provides short-term therapy, group therapy, or patient support groups) | 1 🔾 | 2 🔾 | 3 🔾 |
| b. IBD surgical specialists (e.g., Colorectal surgeon) | 1 🔾 | 2 🔾 | 3 🔾 |
| c. Nutritionist/dietitian | 1 🔾 | 2 🔾 | 3 🔾 |
| d. Ostomy clinic | 1 🔾 | 2 🔾 | 3 🔾 |
| e. Pharmacist to help with IBD medication | 1 🔾 | 2 🔾 | 3 🔾 |
| f. Patient liaison or other staff who can help navigate insurance (e.g., prior authorizations), prescription renewals, and other paperwork | 1 🔾 | 2 🔾 | 3 🔾 |
| g. Alternative medicine (e.g., Pilates, acupuncture, reiki, or yoga to help with stress management) | 1 🔾 | 2 🔾 | 3 🔾 |
| h. Clinical trials | 1 🔾 | 2 🔾 | 3 🔾 |

**C_B4.** In the last 12 months, **when the person with IBD needed care right away**, how often did they get care as soon as they needed?

1 🔾 Never

2 🔾 Sometimes

3 🔾 Usually

4 🔾 Always

5 🔾 Not applicable – They did not need care right away in the past 12 months

**C_B5.** There are many reasons people delay getting medical care. During the past 12 months, did you or the person with IBD **delay or not get medical care** for their IBD for any of the following reasons?

MARK ONLY ONE PER ROW

|  | YES | NO | NOT APPLICABLE OR PERSON WITH IBD DOES NOT HAVE INSURANCE |
| --- | --- | --- | --- |
| a. Difficulty finding a doctor, healthcare professional, clinic, or hospital that accepts their insurance | 1 🔾 | 2 🔾 | 3 🔾 |
| b. An appointment wasn’t available when they needed it | 1 🔾 | 2 🔾 | 3 🔾 |
| c. Couldn’t get to the doctor’s office or clinic when it was open | 1 🔾 | 2 🔾 | 3 🔾 |
| d. It takes too long to get to the doctor’s office or clinic | 1 🔾 | 2 🔾 | 3 🔾 |
| e. Too busy with work or other commitments to take the time | 1 🔾 | 2 🔾 | 3 🔾 |
| f. Couldn’t get through to the office or clinic on the telephone | 1 🔾 | 2 🔾 | 3 🔾 |
| g. Didn’t have transportation | 1 🔾 | 2 🔾 | 3 🔾 |
| h. Waiting for insurance approval | 1 🔾 | 2 🔾 | 3 🔾 |
| 1. Other (*specify*) | 1 🔾 | 2 🔾 | 3 🔾 |

MEDICATIONS, TESTS, AND TREATMENTS

The next questions are about medications for IBD. When answering these questions, think about the medications prescribed for the person with IBD by their IBD healthcare professional. Diet, nutrient rich formula, and exclusive enteral nutrition (EEN) are not considered medications in this section.

**C_C1.** Think about the medications the person with IBD takes to manage their IBD. Please consider both new and renewed prescriptions that are a part of their regular treatment or that they take occasionally to treat their symptoms.

In the last 12 months, was the person with IBD prescribed **any medication** for their IBD?

1 🔾 Yes

2 🔾 No

GO TO C_C8

3 🔾 Don’t know

| **Programmer Box** |
| --- |
| - IF C_C1 = 1, go to C_C2. - If C_c1 = 2 or 3, go to C_C8. |

**C_C2.** Corticosteroids suppress the immune system. Corticosteroids include medications such as Prednisone (Deltasone®), Prednisolone (Oraped®, Prelone®, Pediapred®), Methylprednisolone (A-Methapred®, Depo-Medrol®, Medrol Dosepak®, Solu-Medrol®), and Budesonide (Entocort® EC, UCERIS™).

In the last 12 months, has the person with IBD taken **corticosteroids** for their IBD?

1 🔾 Yes

2 🔾 No

GO TO C_C3

3 🔾 Don’t know

| **Programmer Box** |
| --- |
| - IF C_c2 = 1, go to C_C2a. - If C_c2 = 2 or 3, go to C_C3. |

**C_C2a.** In the last 12 months, what is the **longest amount of time** the person with IBD has taken corticosteroids **daily** for their IBD?

1 🔾 They did not take corticosteroids daily

2 🔾 Every day for less than 2 weeks

3 🔾 Every day for 2 to 4 weeks

4 🔾 Every day for more than 4 weeks, but less than 12 weeks

5 🔾 Every day for 12 weeks or more

**C_C3.** Biologic therapies are bioengineered drugs that target very specific molecules involved in the inflammatory process. Biosimilars are similar, near identical copies of already approved biologic therapies. Biologic therapies and biosimilars are given as an infusion or an injection.

Examples of biologic therapies and biosimilars include Adalimumab [including biosimilars] (Humira®, Cyltezo™, Hyrimoz™, Abrilada™, Amjevita®, HADLIMA, Hulio®), Certolizumab pegol (Cimzia®), Golimumab (Simponi®) and Infliximab [including biosimilars and unbranded] (Remicade®, Renflexis®, Avsola™, Inflectra™, IXIFI™), Natalizumab (Tysabri®), Risankizumab (Skyrizi®), Ustekinumab (Stelara®), and Vedolizumab (Entyvio®).

Targeted synthetic small molecules help reduce inflammation by specifically targeting parts of the immune system that play a role in inflammation. They are taken orally in pill form. There are three targeted synthetic small molecules approved to treat ulcerative colitis – Ozanimod (Zeposia®), Tofacitinib (Xeljanz®), Upadacitinib (RINVOQ®).

In the last 12 months, has the person with IBD taken **a biologic therapy, biosimilar, or targeted synthetic small molecule** for their IBD?

1 🔾 Yes

2 🔾 No

3 🔾 Don’t know

**C4.**  Sometimes patients have difficulties obtaining their IBD prescription medications. In the past 12 months, did you or the person with IBD experience any of the following issues?

MARK ONLY ONE PER ROW

|  | YES | NO | NOT APPLICABLE OR PERSON WITH IBD DOES NOT HAVE INSURANCE |
| --- | --- | --- | --- |
| a. Insurance would not cover a medication prescribed by their IBD healthcare professional | 1 🔾 | 2 🔾 | 3 🔾 |
| b. Insurance would not cover the medication dose or frequency prescribed by their IBD healthcare professional | 1 🔾 | 2 🔾 | 3 🔾 |
| c. Had to wait more than 2 days to fill a prescription because the pharmacy needed an additional approval from their insurance | 1 🔾 | 2 🔾 | 3 🔾 |
| d. Insurance requires that they try a different medication before the preferred medication prescribed by their IBD healthcare professional | 1 🔾 | 2 🔾 | 3 🔾 |
| e. Spent more than 2 hours on the phone with their insurance provider trying to get a prescription covered | 1 🔾 | 2 🔾 | 3 🔾 |
| f. Could not receive an infusion at their preferred location | 1 🔾 | 2 🔾 | 3 🔾 |
| g. Had to take time off from work to be available to receive medication supplies at home | 1 🔾 | 2 🔾 | 3 🔾 |
| h. Sought out a second opinion or made an appointment with another healthcare professional to assist with a denied insurance appeal | 1 🔾 | 2 🔾 | 3 🔾 |
| i. Experienced clerical errors (e.g., prescription sent to wrong pharmacy, incorrect dosage provided, pharmacy could not find script, etc.) | 1 🔾 | 2 🔾 | 3 🔾 |
| j. Decided not to fill a prescription because of possible side effects | 1 🔾 | 2 🔾 | 3 🔾 |

**C_C5.** Think about the medications the person with IBD takes to manage their IBD. Please consider both new and renewed prescriptions that are a part of their regular treatment or that they take occasionally to treat their symptoms.

In the last 12 months, was the person with IBD able to get **all** of their IBD prescription medication?

1 🔾 Yes GO TO C_C6

2 🔾 No

3 🔾 Don’t know

| **Programmer Box** |
| --- |
| - IF C_c5 = 1, go to C_c6. - If C_c5 = 2 OR 3, go to C_C5a. |

**C_C5a.** In the last 12 months, how often did the person with IBD get the **prescription medication** they needed to treat their IBD?

1 🔾 Never

2 🔾 Sometimes

3 🔾 Usually

4 🔾 Always

**C_C6.** In the past 12 months, what was the **longest delay** the person with IBD experienced waiting for **insurance to approve** their prescription medications to treat their IBD?

1 🔾 Less than 1 week

2 🔾 1 week

3 🔾 2 to 3 weeks

4 🔾 1 to 3 months

5 🔾 4 or more months

6 🔾 They never received their medications

7 🔾 Not applicable – They did not experience a delay in the past 12 months

8 🔾 Not applicable – They do not have health insurance

**C_C6b.** In the last 12 months, if they were **unable** to get their IBD medication or their IBD medication was **delayed**, did they experience any of the following?

Mark all that apply

1 □ Unable to work or attend school

2 □ Unable to do daily activities (e.g., cooking, caring for family)

3 □ Decreased quality of life (e.g., decreased appetite, disturbed sleep, negative impact on mental health)

4 □ Increased pain

5 □ Took a nonsteroid medication to treat symptoms (e.g., anti-diarrheal medication, such as Imodium/loperamide or pain medication, such as Norco, Tylenol, Tramadol)

6 □ Took steroid/corticosteroid to treat symptoms (e.g., Prednisone)

7 □ A new flare

8 □ Worsening of an existing flare

9 □ An abscess, fistula, or infection

10 □ Emergency department visit

11 □ Hospitalization

12 □ Surgery to treat their IBD

13 □ They did not experience any adverse events

14 □ Other *(specify)* _____________________________________________________________

NA 🔾 They were able to get all of their medications and did not experience a delay

The next questions are about tests and treatments the person with IBD needs for their IBD care.

**C_C7.**  Therapeutic drug monitoring is testing that measures the amount of medication or the presence of anti-drug antibodies in your blood. This type of testing is often used when a patient is on biological therapies.

In the last 12 months, did the person with IBD need **therapeutic drug monitoring**?

1 🔾 Yes

2 🔾 No

GO TO C_C8

3 🔾 Don’t know

| **Programmer Box** |
| --- |
| - IF C_C7 = 1, go to C_C7a. - If C_c7 = 2 or 3, go to C_c8. |

**C_C7a.** In the last 12 months, how often was the person with IBD able to get **insurance to cover** their therapeutic drug monitoring?

If the person with IBD has had more than one therapeutic drug monitoring test in the past 12 months, consider all and select the answer that best reflects their general experience.

1 🔾 Never

2 🔾 Sometimes

3 🔾 Usually

4 🔾 Always

5 🔾 Unsure – it is still under review by my insurance

6 🔾 Not applicable – They do not have health insurance

**C_C8.** A calprotectin stool test is used to measure calprotectin - a protein in stool that indicates inflammation in the intestines. Healthcare professionals may use this test to monitor levels of inflammation and inform treatment.

In the last 12 months, did the person with IBD need a **calprotectin stool test**?

1 🔾 Yes

2 🔾 No

GO TO C_C9

3 🔾 Don’t know

| **Programmer Box** |
| --- |
| - IF C_c8 = 1, go to C_c8a. - If C_c8 = 2 or 3, go to C_c9. |

**C_C8a.** In the last 12 months, how often was the person with IBD able to get **insurance to cover** their calprotectin stool test(s)?

If the person with IBD has had more than one stool test in the past 12 months, consider all and select the answer that best reflects their general experience.

1 🔾 Never

2 🔾 Sometimes

3 🔾 Usually

4 🔾 Always

5 🔾 Unsure – it is still under review by my insurance

6 🔾 Not applicable – They do not have health insurance

**C_C9.**  In the last 12 months, did the person with IBD need **other tests** for their IBD care?

Tests may include blood and stool tests, endoscopic procedures (e.g., colonoscopy, upper endoscopy, sigmoidoscopy, capsule endoscopy), radiology scans, and diagnostic imaging (e.g., X-rays, upper GI scans, CT scans, MRE).

1 🔾 Yes

2 🔾 No

GO TO C_C10

3 🔾 Don’t know

| **Programmer Box** |
| --- |
| - IF C_c9 = 1, go to C_c9a. - If C_c9 = 2 or 3, go to C_c10. |

**C_C9a.** In the last 12 months, how often was the person with IBD able to get **insurance to cover** their other test(s)?

If the person with IBD has had more than one test in the past 12 months, consider all and select the answer that best reflects their general experience.

1 🔾 Never

2 🔾 Sometimes

3 🔾 Usually

4 🔾 Always

5 🔾 Unsure – it is still under review by my insurance

6 🔾 Not applicable – They do not have health insurance

**C_C10.** In the last 12 months, did the person with IBD need **other treatments** for their IBD care?

**Other treatments** may include surgery (e.g., colectomy, proctocolectomy), treatment for abscesses and fistulas (e.g., antibiotics, surgery), and treatment for extraintestinal complications of IBD (e.g., iron infusions for anemia, supplements for osteoporosis). Please exclude all IBD medications from your answer.

1 🔾 Yes

2 🔾 No

GO TO C_D1

3 🔾 Don’t know

| **Programmer Box** |
| --- |
| - IF C_c10 = 1, go to C_C10A. - If C_c10 = 2 or 3, go to C_D1. |

**C_C10a.**In the last 12 months, how often was the person with IBD able to get **insurance to cover** their other treatment(s)?

If the person with IBD has had more than one treatment in the past 12 months, consider all and select the answer that best reflects their general experience.

1 🔾 Never

2 🔾 Sometimes

3 🔾 Usually

4 🔾 Always

5 🔾 Unsure – it is still under review by my insurance

6 🔾 Not applicable – They do not have health insurance

FINANCIAL BARRIERS

The next set of questions are about paying for IBD healthcare. This includes bills for healthcare professionals, hospitals, therapists, medication, equipment, and nursing home or home care.

**C_D1.** In the past 12 months, did you or the person with IBD have trouble paying (e.g., late paying a bill, had to wait for your next paycheck) or were unable to pay for any medical bills related to their IBD care?

1 🔾 Yes

2 🔾 No

3 🔾 Don’t know

**C_D2.** In order to pay their healthcare or insurance costs related to their IBD, did you or the person with IBD do any of the following in the past 12 months?

MARK ONLY ONE PER ROW

|  | YES | NO | DON’T’ KNOW |
| --- | --- | --- | --- |
| a. Borrowed money from friends or family | 1 🔾 | 2 🔾 | 3 🔾 |
| b/c.Took out any type of loan (e.g., an additional mortgage)/ Sought the aid of a charity or non-profit organization | 1 🔾 | 2 🔾 | 3 🔾 |
| d. Increased credit card debt | 1 🔾 | 2 🔾 | 3 🔾 |
| e. Cut back on food, clothing, or basic household items | 1 🔾 | 2 🔾 | 3 🔾 |
| f. Put off vacations or major household purchases | 1 🔾 | 2 🔾 | 3 🔾 |
| g. Took money out of retirement, college, or other long-term savings account | 1 🔾 | 2 🔾 | 3 🔾 |
| h. Took an extra job or worked more hours | 1 🔾 | 2 🔾 | 3 🔾 |
| i. Changed their living situation (e.g., moving in with family or friends) | 1 🔾 | 2 🔾 | 3 🔾 |
| j. Used up all or most of your/their savings | 1 🔾 | 2 🔾 | 3 🔾 |
| k. Used the internet or social media to raise funds to pay for medical care | 1 🔾 | 2 🔾 | 3 🔾 |

**C_D3.** During the past 12 months, were any of the following true for the person with IBD regarding their IBD medication?

Mark all that apply

1 □ They did not get their medication because of the cost

2 □ They skipped medication doses to save money

3 □ They took less medication to save money

4 □ They delayed filling a prescription to save money

5 □ They asked their doctor for a lower cost medication to save money

6 □ They bought medication from another country to save money

7 □ They used alternative therapies to save money

8 □ Other *(specify)*

NA 🔾 Cost did not interfere with my ability to get the medication

| **Programmer Box** |
| --- |
| - IF c_D3 = 1 – 8, go to c_D4. - If c_D3 = NA, go to c_D5. |

**C_D4.** During the past 12 months, when the person with IBD **did not take their IBD medication as prescribed**, did they experience any of the following?

Mark all that apply

1 □ Unable to work or attend school

2 □ Unable to do daily activities (e.g., cooking, caring for family)

3 □ Decreased quality of life (e.g., decreased appetite, disturbed sleep, negative impact on mental health)

4 □ Increased pain

5 □ Took a nonsteroid medication to treat symptoms (e.g., anti-diarrheal medication, such as Imodium/loperamide or pain medication, such as Norco, Tylenol, Tramadol)

6 □ Took steroid/corticosteroid to treat symptoms (e.g., Prednisone)

7 □ A new flare

8 □ Worsening of an existing flare

9 □ An abscess, fistula, or infection

10 □ Emergency department visit

11 □ Hospitalization

12 □ Surgery to treat their IBD

13 □ Other *(specify)*

NA 🔾 They did not experience any adverse events

**C_D5.** Coupons, copay cards, and copay assistance programs help patients with health insurance afford expensive prescription medications. Patient assistance programs offer free or low-cost prescription medications to people who do not have insurance or who have limited health insurance that covers only part of their healthcare expenses.

In the past 12 months, has the person with IBD **received a discount on a medication**, either through a coupon, a copay card, a drug company patient assistance program, or some other kind of medication discount?

1 🔾 Yes

2 🔾 No

GO TO C_E1

3 🔾 Don’t know

| **Programmer Box** |
| --- |
| - IF C_D5 = 1, go to C_D5a. - If C_D5 = 2 or 3, go to C_E1. |

**D5a.** Did the person with IBD **use up or run out of one or more co-pay assistance(s)** before the end of the year (e.g., manufacturer payments were no longer applied to their deductible or out-of-pocket expenses)?

1 🔾 Yes

2 🔾 No

3 🔾 Don’t know

EXCLUSIVE ENTERAL NUTRITION (EEN)

| **Programmer Box** |
| --- |
| - IF SURVEY TYPE IS CAREGIVER AND C_A2 = 1, go to section e. Passed primary crohns disease screening. - IF SURVEY TYPE IS CAREGIVER AND C_A2 does not equal 1, GO TO SECTION F. failed primary crohns disease screening. |

The next questions are about treating Crohn's disease with exclusive enteral nutrition (EEN), a nutrient rich formula used to provide all of a patient's nutrition. Patients drink the enteral formula or ingest it through a feeding tube (e.g., nasogastric (NG) tube, nasoduodenal (ND) tube, nasojejunal (NJ) tube, gastronomy tube (G-tube), or jejunostomy tube (J-tube)). Common formulas include Boost, Ensure, Orgain, Vital, Peptamen, and Modulen.

**C_E1.** In the last 12 months, did the person with IBD’s IBD healthcare professional prescribe exclusive enteral nutrition (EEN) or nutrient rich formula to treat their IBD?

1 🔾 Yes, through a tube (e.g., NG tube, ND tube, NJ tube, G-tube, or J-tube)

2 🔾 Yes, by mouth (e.g., oral or drink it)

3 🔾 No

GO TO C_F1

4 🔾 Don’t know

| **Programmer Box** |
| --- |
| - IF C_E1 = 1, 2, go to C_E2. - If C_E1 = 3 or 4, go to C_F1. |

For the remainder of this section, we will refer to exclusive enteral nutrition (EEN) and nutrient rich formula as “formula”.

**C_E2.**  Does the person with IBD’s insurance pay for their formula?

1 🔾 Yes, insurance covers all of the costs

2 🔾 Yes, but insurance only covers part of the costs

3 🔾 No, insurance does not cover any of the costs

4 🔾 Unsure – it is still under review by my insurance

5 🔾 Not applicable – They do not have health insurance

**C_E3.** In the last 12 months, were any of the following true for the person with IBD regarding their formula?

Mark all that apply

1 □ They did not get their formula because of the cost

2 □ They skipped formula doses to save money

3 □ They took less formula to save money

4 □ They delayed filling a formula prescription to save money

5 □ They asked their doctor for a lower cost formula to save money

6 □ They bought formula from another country to save money

7 □ They tried a medication covered by insurance instead

8 □ They tried a different formula covered by their insurance instead of taking the one prescribed by their healthcare professional

9 □ They did not start formula

NA & 10 🔾They had access to the prescribed formula and took it as instructed

11 □Other *(specify)*

| **Programmer Box** |
| --- |
| - IF C_E3 = 1, 2, 3, 4, 5, 6, 7, 8, 9, OR 11, go to C_E4. - If C_E3 = na or 10, go to C_F1. |

**C_E4.**  During the time the person with IBD could not get or did not take their formula as prescribed, did they experience any of the following with regards to their IBD?

Mark all that apply

1 □ Unable to work or attend school

2 □ Unable to do daily activities (e.g., cooking, caring for family)

3 □ Decreased quality of life (e.g., decreased appetite, disturbed sleep, negative impact on mental health)

4 □ Increased pain

5 □ Took a nonsteroid medication to treat symptoms (e.g., anti-diarrheal medication, such as Imodium/loperamide or pain medication, such as Norco, Tylenol, Tramadol)

6 □ Took steroid/corticosteroid to treat symptoms (e.g., Prednisone)

7 □ A new flare

8 □ Worsening of an existing flare

9 □ An abscess, fistula, or infection

10 □ Emergency department visit

11 □ Hospitalization

12 □ Surgery to treat their IBD

13 □ Other *(specify)*

NA 🔾 They did not experience any adverse events

LEGISLATION AND ADVOCACY

| **Programmer Box** |
| --- |
| - IF C_A5 = one of the following states is selected: California, Oregon, Washington, Arizona, New Mexico, Colorado, South Dakota, Nebraska, Kansas, Oklahoma, Texas, Louisiana, Arkansas, Missouri, Iowa, Minnesota, Wisconsin, Illinois, Kentucky, Indiana, Tennessee, Mississippi, Ohio, Georgia, West Virginia, Virginia, North Carolina, Maryland, Delaware, Connecticut, Massachusetts, New York, Pennsylvania, and Maine, go to section F. Passed step therapy state screening. - IF C_A5 = one of the following states is selected: Nevada, Utah, Wyoming, Montana, Vermont, New Hampshire, Idaho, North Dakota, South Carolina, Alabama, Florida, New Jersey, Alaska, Hawaii, Rhode Island, Michigan and DC, GO TO SECTION g. failed step therapy state screening. |

Earlier you indicated that the person with IBD lives in [FILL STATE FROM C_A5]. The next question is about IBD legislation specific to this state.

**C_F1.** Step therapy, also known as “fail first,” is an insurance process which requires patients to try one or more medications, typically a generic or lower cost medicine, to treat a health condition. Patients must fail these medication(s) before allowing a “step up” to another medicine that may be more expensive for the insurer. **The person with IBD lives in a state that has passed legislation** mandating changes to this insurance practice.

How familiar are you or the person with IBD, if at all, with their state’s legislation to change step therapy or fail first protocols?

1 🔾 Very familiar

2 🔾 Somewhat familiar

3 🔾 Heard of, but know very little

4 🔾 Not at all familiar

AWARENESS OF FOUNDATION AND OTHER TOOLS

**C_G1.** Below is a list of resources used by people with IBD and their caregivers when they are having trouble getting medical care (e.g., getting insurance approval to see an IBD specialist or denial of a test or medication their IBD healthcare professional ordered).

Which of the following resources, if any, are you or the person with IBD aware of or have used?

MARK ONLY ONE PER ROW

|  | NOT AWARE OF | AWARE OF | HAVE USED | NOT APPLICABLE OR PERSON WITH IBD DOES NOT HAVE INSURANCE |
| --- | --- | --- | --- | --- |
| a. IBD Insurance Checklist (i.e., a guide to evaluate and compare insurance plans) from the Crohn’s & Colitis Foundation | 1 🔾 | 2 🔾 | 3 🔾 | 4 🔾 |
| b. Tool to search patient financial assistance programs from the Crohn’s & Colitis Foundation | 1 🔾 | 2 🔾 | 3 🔾 | 4 🔾 |
| c. Customizable appeal letters (e.g., template letter if denied medication, or treatment) from the Crohn’s & Colitis Foundation | 1 🔾 | 2 🔾 | 3 🔾 | 4 🔾 |
| d. IBD Help Center from the Crohn’s & Colitis Foundation | 1 🔾 | 2 🔾 | 3 🔾 | 4 🔾 |
| e. In-person and online support groups | 1 🔾 | 2 🔾 | 3 🔾 | 4 🔾 |
| f. Resources from other patient advocacy or non-profit organizations | 1 🔾 | 2 🔾 | 3 🔾 | 4 🔾 |
| g. Resources from pharmaceutical companies (e.g., website and mailings with information about understanding insurance and financial assistance, nurse or videos to train patient in administering medication, and nurse ambassadors/hotlines) | 1 🔾 | 2 🔾 | 3 🔾 | 4 🔾 |
| h. Employee assistance programs (EAP) (e.g., an employee benefit that can include assessments, counseling, and referrals for additional services) | 1 🔾 | 2 🔾 | 3 🔾 | 4 🔾 |
| i. Other (*specify*) | 1 🔾 | 2 🔾 | 3 🔾 | 4 🔾 |

**C_G2.** Think about the most recent time the person with IBD had difficulties getting IBD care (e.g., insurance troubles, understanding medical bills, getting a healthcare appointment), did you or the person with IBD reach out to family or friends for assistance?

1 🔾 Yes

2 🔾 No

3 🔾 Not applicable – They have not experienced any difficulties

**C_G3.** Please indicate how confident you are in the following statements about the person with IBD’s care:

MARK ONLY ONE PER ROW

|  | NOT CONFIDENT AT ALL | SLIGHTLY CONFIDENT | FAIRLY CONFIDENT | VERY CONFIDENT | NOT APPLICABLE OR PERSON WITH IBD DOES NOT HAVE INSURANCE |
| --- | --- | --- | --- | --- | --- |
| a. I know what to do if their insurance refuses to pay for a service I think should be covered | 1 🔾 | 2 🔾 | 3 🔾 | 4 🔾 | 5 🔾 |
| b. I know what questions to ask their insurance if they have a coverage problem | 1 🔾 | 2 🔾 | 3 🔾 | 4 🔾 | 5 🔾 |
| c. I know what to do if their insurance requires them to start and fail a different medication before allowing them to take the medication their IBD healthcare professional ordered | 1 🔾 | 2 🔾 | 3 🔾 | 4 🔾 | 5 🔾 |
| d. Their IBD healthcare professional could help get insurance approval for a denied medication | 1 🔾 | 2 🔾 | 3 🔾 | 4 🔾 | 5 🔾 |
| e. I/They can get insurance approval in a timely manner | 1 🔾 | 2 🔾 | 3 🔾 | 4 🔾 | 5 🔾 |

**C_G4.**  What other resources would be helpful when the person with IBD is having trouble getting medical care, including medications?

Mark all that apply

1 □ Appeals process visual or graphic designed to show patients steps in the appeals process included expected timing and role of the patient

2 □ Suggested conversation starters to help patients feel more confident when discussing their care with their healthcare professional

3 □ Step-by-step brief videos describing what to do when denied coverage by their insurance company

4 □ A “Navigating Appeals” resource webpage for patients on the Crohn’s & Colitis Foundation website

5 □ Social sharing campaign for patients to share appeal successes

6 □ Other ideas (*specify*)

dk 🔾 Don’t know

YOUR EXPERIENCE

**C_H1.** Please use this space to share anything about experiences accessing healthcare for IBD that we have not asked about already and that it is important for us to know.

DEMOGRAPHIC CHARACTERISTICS AND CONTACT INFORMATION

The final set of questions asks about background of the person with IBD. Answers to these questions will be used to describe the type of people in the survey.

**C_I1.** What year was the person with IBD were diagnosed with IBD?

_____________ [Dropdown]

| **Programmer Box** |
| --- |
| - drop down RANGE IS 2023 – … 2000; Before 2000; Don’t remember when they were diagnosed; prefer not to disclose |

**C_I2.**  Which of the following best describe their IBD over the **past 6 months**?

1 🔾 Constantly active, giving them symptoms every day

2 🔾 Often active, giving them symptoms most days

3 🔾 Sometimes active, giving them symptoms on some days

4 🔾 Occasionally active, giving them symptoms 1-2 days a month

5 🔾 Rarely active, giving them symptoms only a few days in the past 6 months

6 🔾 They were well in the past 6 months, what they consider a remission or absence of symptoms

7 🔾 Don’t know

**C_I3.**  Is the person with IBD currently covered by any of the following types of health insurance or health coverage plans?

MARK ALL THAT APPLY

1 🔾 Not covered by health insurance

2 □ Employer or Union based insurance

3 □ Indian Health Service

4 □ Individual or Small Group Plan (e.g., through a state exchange)

5 □ Medicare

6 □ Medicaid, Medical Assistance, or any kind of non-Medicare government-assistance plan

7 □ Military health care (e.g., TRICARE, VA, CHAMP-VA)

8 □ Single service plan (e.g., dental, vision, prescriptions)

9 □ CHIP (SCHIP/Children's Health Insurance Program)

10 🔾 Prefer not to disclose

11 🔾 Don’t know

12 □ Other (*specify*) ________________________________________________________________

**C_I4.**  Are they of Hispanic, Latino/a, or Spanish origin?

1 🔾 Yes, Hispanic, Latino/a, or Spanish origin

2 🔾 No, not of Hispanic, Latino/a, or Spanish origin

3 🔾 Prefer not to disclose

4 🔾 Don’t know

**C_I5.** What is their race?

MARK ALL THAT APPLY

1 □ American Indian or Alaska Native

2 □ Asian

3 □ Black or African American

4 □ Native Hawaiian or Other Pacific Islander

5 □ White

6 🔾 Prefer not to disclose

7 🔾 Don’t know

8 □ Other self-identities (*specify*) _______________________________________________________

**C_I6.** Do they identify as Middle Eastern or North African?

1 🔾 Yes

2 🔾 No

3 🔾 Prefer not to disclose

0 🔾 Don’t know

**C_I7.** What sex were they assigned at birth, on their original birth certificate?

1 🔾 Female

2 🔾 Male

3 🔾 Prefer not to disclose

0 🔾 Don’t know

**C_I8.** What is their current gender?

1 🔾 Female

2 🔾 Male

3 🔾 Transgender

4 🔾 Prefer not to disclose

5 🔾 Don’t know

0 🔾 They use a different term:

**C_I9.** What is the highest degree or level of school that they have completed?

1 🔾 Some school, but no high school diploma

2 🔾 High school diploma or GED

3 🔾 Some college credit, but no degree

4 🔾 Associate’s degree (e.g., AA, AS)

5 🔾 Bachelor’s degree (e.g., BA, BS)

6 🔾 Master’s degree or higher (e.g., MA, MS, PhD, MD, DO)

7 🔾 Prefer not to disclose

8 🔾 Don’t know

0 🔾 Other *(specify)* __________________________________________________________________

**C_I10.** What is their current employment status?

MARK ALL THAT APPLY

1 □ Employed, full time

2 □ Employed, part time

3 □ Unemployed

4 □ Homemaker

5 □ Student

6 □ Retired

7 □ Unable to work

8 🔾 Prefer not to disclose

9 🔾 Don’t know

**C_I11.**  What is their home ZIP code?

| | | | | |

**C_I12.** Would you like to take this survey again on behalf of another person with IBD that you care for?

1 🔾 Yes, I would like to take the survey for another person in my care GO TO A1 OF CAREGIVER SURVEY

2 🔾 No, I do not want to repeat the survey

GO TO END

3 🔾 No, I already completed the survey for all persons in my care

| **Programmer Box** |
| --- |
| - IF C_I12 = 1, go to page below – link to caregiver survey - If c_i12 = 2 or 3, go to THANK YOU. |
